# A Climatology of Long‐Duration High 2‐MeV Electron Flux Periods in the Outer Radiation Belt

**DOI:** 10.1029/2022JA030661

**Published:** 2022-08-15

**Authors:** D. Mourenas, O. V. Agapitov, A. V. Artemyev, X.‐J. Zhang

**Affiliations:** ^1^ CEA DAM DIF Arpajon France; ^2^ Laboratoire Matière en Conditions Extrêmes Paris‐Saclay University CEA Bruyères‐le‐Châtel France; ^3^ Space Sciences Laboratory University of California Berkeley CA USA; ^4^ Department of Earth, Planetary, and Space Sciences University of California Los Angeles CA USA

**Keywords:** radiation belt, electron flux, space weather, electron acceleration, return levels, forecast

## Abstract

Since the advent of the Space Age, the importance of understanding and forecasting relativistic electron fluxes in the Earth’s radiation belts has been steadily growing due to the threat that such particles pose to satellite electronics. Here, we provide a model of long‐duration periods of high time‐integrated 2‐MeV electron flux deep inside the outer radiation belt, based on the significant correlation obtained in 2001–2017 between time‐integrated electron flux measured by satellites and a measure of the preceding time‐integrated homogenized *aa*_*H*_ geomagnetic index. We show that this correlation is likely due to a stronger cumulative chorus wave‐driven acceleration of relativistic electrons and a stronger cumulative inward radial diffusion of such electrons during periods of higher time‐integrated geomagnetic activity. Return levels of 2‐MeV electron flux are provided based on Extreme Value analysis of time‐integrated geomagnetic activity over 1868–2017, in rough agreement with estimates based on 20‐year data sets of measured flux. A high correlation is also found between our measure of time‐integrated geomagnetic activity averaged over each solar cycle and averaged sunspot numbers, potentially paving the way for forecasts of time‐integrated relativistic electron flux during future solar cycles based on predictions of solar activity.

## Key Points

- A predictive model of high time‐integrated 2‐MeV electron flux is provided based on correlations with time‐integrated *Int*(*aa*_*H*_)
- Return levels of relativistic electron flux are estimated based on Extreme Value analysis of strong time‐integrated *Int*(*aa*_*H*_) events
- High correlations between solar cycle averaged *Int*(*aa*_*H*_) and sunspot numbers might allow forecasts during future solar cycles

## 1 Introduction

The ever‐growing reliance of modern technological society on space‐based connections and services in many economic and military sectors (e.g., internet, financial transactions, navigation, various control systems) has increased hazards associated with potential failures of Global Navigation Satellite Systems and other satellites in the aftermath of strong solar wind disturbances (Eastwood et al., 2017; Glauert et al., 2018; Meredith et al., 2015; Riley et al., 2018). Indeed, electronic devices inside satellites are particularly at risk from the total ionizing dose (TID) of radiation, corresponding to direct or indirect ionization of a semiconductor by incident relativistic electrons in the Earth's radiation belts (Cochran et al., 2009; Zheng et al., 2019), which can lead to satellite anomalies and sometimes even total failure (Chen et al., 2021; Ecoffet, 2013; Iucci et al., 2005). It is worth emphasizing that this TID or radiation absorbed dose (in rad or Gray units) is a long‐term cumulative effect, causing a gradual degradation of semiconductor performance (Chen et al., 2021; Stassinopoulos & Raymond, 1988; Zheng et al., 2019).

Spacecraft electronic devices are usually protected from TID by an aluminum shielding of ∼100–300 mil thickness (corresponding to 2.5–7.5 mm or ∼0.7–2 g/cm^2^), which strongly reduces the penetrating electron flux at energies below ∼1–2 MeV (Chen et al., 2021; Stassinopoulos & Raymond, 1988). Therefore, the main TID risk for satellites is due to long‐duration periods (typically days to weeks) of high relativistic (>1–2 MeV) electron flux, which correspond to the periods of highest time‐integrated electron flux (also called fluence; e.g., see Chen et al., 2021). Such periods represent a much bigger TID threat than the much shorter‐lived periods of highest instantaneous (or hourly or daily) electron flux. Relativistic electron fluxes represent a particularly important hazard for spacecraft electronics at altitudes comprised between 20,000 and 40,000 km in the outer radiation belt, where geomagnetic storms or high‐speed solar wind streams can trigger prolonged periods of elevated 2‐MeV electron flux (Baker et al., 1990; Glauert et al., 2018; Iucci et al., 2005; Li & Hudson, 2019; Mourenas, Artemyev, & Zhang, 2019; Murphy et al., 2018; Ozeke et al., 2020; Thorne et al., 2013). Such long‐duration (typically ∼10 days at *L* ∼ 4.5), high 2‐MeV electron flux periods have been found to be the main contributors to the total yearly time‐integrated electron flux during active years (Mourenas, Artemyev, & Zhang, 2019) and, therefore, to the TID in satellites.

Assessing radiation hazards in the heart of the outer radiation belt (around *L* ∼ 4.5) is important for Global Positioning System (GPS) satellites, but also for spacecraft on geostationary transfer orbit (GTO) using slow electric orbit raising, which spend months in this region (Glauert et al., 2018). Various models have been developed to forecast MeV electron fluxes up to ∼1–2 days ahead based on the past history of geomagnetic indices and/or solar wind parameters, often with additional inputs such as the measured electron flux at the same or lower energy during the preceding days (Boynton, Balikhin, et al., 2016; Chu et al., 2021; Glauert et al., 2021; Pires de Lima et al., 2020). In the present study, we develop a predictive model of long‐duration 10‐day periods of high time‐integrated 2‐MeV electron flux based on the past time‐integrated geomagnetic activity, similar to our previous models relying on *AE* and *ap* indices (Mourenas, Artemyev, & Zhang, 2019), but using now the homogenized *aa* index, denoted *aa*_*H*_, continuously available from 1868 to 2017 (Lockwood, Chambodut, Barnard, Owens, & Clarke, 2018; Lockwood, Chambodut, Barnard, Owens, Clarke, & Mendel, 2018). The *aa* and *aa*_*H*_ indices are based on the range of variation of the horizontal component of the geomagnetic field (Mayaud, 1980).

The proposed model is developed in Section 2 based on Van Allen Probes electron flux data in 2012–2017 (Baker et al., 2013) and GPS satellite data in 2003–2005 (Morley et al., 2016), and its performance is tested using various metrics (Zheng et al., 2019). A key advantage of the proposed model is that it can approximately predict both the start of each 10‐day period of high 2‐MeV electron flux and the total time‐integrated electron flux that will be accumulated over these next 10 days. Therefore, it can provide 1‐day‐ahead to 7‐day‐ahead warnings of a high TID based on the past history of only one ground‐based geomagnetic index, allowing satellites operators to take temporary measures to prevent damage. In contrast, other forecasting models usually predict electron flux at most 1–2 days in advance and often need additional inputs from satellites (e.g., see Boynton, Balikhin, et al., 2016; Chu et al., 2021; Glauert et al., 2021; Pires de Lima et al., 2020). In essence, the proposed model can be viewed as a very simplified short‐term (10‐day) climatological model of space weather, compared to other forecasting models more similar to meteorological models. However, an inherent drawback of the proposed model is its inability to predict electron flux outside prolonged periods of high time‐integrated flux. A brief analysis of the physical phenomena producing these 10‐day periods of elevated relativistic electron flux is provided at the end of Section 2.

A complementary strategy for mitigating total radiation dose effects on spacecraft is to develop a long‐term climatology of space weather events, allowing satellite designers to harden electronic devices for surviving the strongest events expected during the satellite lifetime (typically 10–20 years). However, the longest data set of 2‐MeV electron flux near *L* ∼ 4.5, obtained from modern particle sensors on GPS satellites, represents less than 2 solar cycles of relatively homogeneous data (Morley et al., 2016), and other missions provide even shorter data sets (e.g., 6 years for the Van Allen Probes). As an alternative to statistics of satellite data, a physics‐based three‐dimensional Fokker‐Planck code has been used to simulate electron flux variations over 30 years (Glauert et al., 2018). Nevertheless, reliable estimates of maximum event strength require a statistical analysis of longer‐duration (>100 years) data sets (Riley et al., 2018).

Accordingly, we analyze in Section 3 the *aa*_*H*_ index available from 1868 to 2017 (Lockwood, Chambodut, Barnard, Owens, & Clarke, 2018; Lockwood, Chambodut, Barnard, Owens, Clarke, & Mendel, 2018) to estimate the return levels of periods of high time‐integrated geomagnetic activity. Next, building on the significant correlation obtained in Section 2 between time‐integrated *aa*_*H*_ and long‐duration periods of high 2‐MeV electron flux, we provide estimates of the return levels of such long‐duration high 2‐MeV electron flux periods. These results are compared with previous estimates based on the available data sets of electron flux. Finally, the high correlation found between time‐integrated *aa*_*H*_ and sunspot numbers is used to estimate secular variations of time‐integrated electron fluxes.

## 2 Parameterization of High Time‐Integrated 2‐MeV Electron Flux Periods as a Function of *Int*(*aa*_*H*_)

### 2.1 Geomagnetic Activity Data

The original *aa* index, based on the range of variation of the horizontal component of the geomagnetic field after subtraction of quiet day variation, has been devised to mimic the *ap* index over a much longer time span starting in 1868 (Mayaud, 1980). It is constructed from *K* indices determined from 3‐hr measurements at two antipodal middle latitude stations in England and Australia (whereas *ap* and *Kp* are based on measurements at 13 stations), normalized to geomagnetic latitudes ±50° (Mayaud, 1980). A recent work has shown that middle latitude indices, such as *ap* and *aa*, should probably be preferred to the more stochastic auroral indices *AE* and *AL* for long‐term reconstruction/forecasting (Mourenas et al., 2020). Therefore, in the present study we make use of the 1868–2017 data set of the homogenized *aa*_*H*_ index (Lockwood, Chambodut, Barnard, Owens, & Clarke, 2018; Lockwood, Chambodut, Barnard, Owens, Clarke, & Mendel, 2018). This homogenized *aa*_*H*_ index has been constructed by correcting individual *aa* values for secular changes in geomagnetic field, for variations in measurement station calibrations, and for asymmetries between hemispheres, and the final *aa*_*H*_ index has been scaled to match the similar *am* index over 2012–2017 (Lockwood, Chambodut, Barnard, Owens, & Clarke, 2018; Lockwood, Chambodut, Barnard, Owens, Clarke, & Mendel, 2018).

We consider here strong events of continuously elevated *aa*_*H*_ index. Integrating *aa*_*H*_ during such events, we obtain a measure *Int*(*aa*_*H*_) of their cumulative strength, similar to the *Int*(*ap*) measure considered in a previous work (Mourenas, Artemyev, & Zhang, 2019), but somewhat different from the *Int*(*Dst*) and *Int*(*AE*) measures corresponding to long‐duration storms and High Intensity Long Duration Continuous AE Activity (HILDCAA) events, respectively (Mourenas, Artemyev, & Zhang, 2019; Mourenas et al., 2018, 2020; Tsurutani et al., 2006). Mourenas, Artemyev, and Zhang (2019) have shown that the 60 10‐day periods of highest time‐integrated 1.8‐MeV electron flux measured in 2013–2017 by the Van Allen Probes (Baker et al., 2013) at adiabatically invariant shell L* ∼ 4.5, have occurred just after (∼0–2 days after) significant *Int*(*ap*) > 800 nT⋅hr events, calculated with an integration threshold *ap* ≥ 15. Mourenas, Artemyev, and Zhang (2019) have further shown that the magnitudes of such electron flux peaks are well correlated with *Int*(*ap*), and that this correlation can be used to hindcast GPS 2‐MeV electron flux at *L* ∼ 4.2–4.4 in 2002–2012, demonstrating its long‐term usefulness. Hereafter, we build on this study by Mourenas, Artemyev, and Zhang (2019), but we parameterize the same 10‐day periods of highest time‐integrated 2‐MeV electron flux by *Int*(*aa*_*H*_) instead of *Int*(*ap*). Since *aa*_*H*_ ∼ 1.7*ap* on average, we use a threshold *Int*(*aa*_*H*_) > 1,400 nT⋅hr, equivalent to the threshold *Int*(*ap*) > 800 nT⋅hr used by Mourenas, Artemyev, and Zhang (2019), to define strong events. As *aa*_*H*_ sometimes decreases below 20 nT when *ap* = 15, we also use a conservatively low integration threshold *aa*_*H*_ ≥ 18 nT, to prevent several strong *Int*(*ap*) events of *ap* ≥ 15 from being split into two weaker *Int*(*aa*_*H*_) events.

### 2.2 Model of High Time‐Integrated Electron Flux

In this section, we investigate the impact of significant *Int*(*aa*_*H*_) > 1,400 nT⋅hr events on time‐integrated electron flux in the heart of the outer radiation belt. We use the daily‐averaged omnidirectional 2.1 MeV electron flux (level 2, release 03) measured by the Relativistic Electron‐Proton Telescope (REPT) instrument on board the Van Allen Probes (Baker et al., 2013), at magnetic latitudes <25° and at an adiabatically invariant coordinate L* ∼ 4.5 calculated using the TS04D (Tsyganenko & Sitnov, 2005) external field model and the International Geomagnetic Reference Field (IGRF) internal field model. This 2.1 MeV electron flux from REPT has been carefully cross‐calibrated using simultaneous Magnetic Electron Ion Spectrometer (MagEIS) electron flux data from the Van Allen Probes (Boyd et al., 2019), making it more reliable than the 1.8 MeV electron flux from REPT used in a previous study (Mourenas, Artemyev, & Zhang, 2019).

To include in our analysis additional data from a more active solar cycle, we also analyze daily‐averaged omnidirectional 2‐MeV electron fluxes measured at *L* = 4.2–4.4 by GPS satellites in 2001–2011 (Morley et al., 2016, 2017). *L* is determined using the T89 (Tsyganenko, 1989) and IGRF models. GPS satellites have near‐circular orbits at 20,200 km altitude, with a period of 12 hr and an inclination of 55°. GPS electron fluxes are provided by the Combined X‐ray and Dosimeter (CXD) instrument developed at Los Alamos National Laboratory, in 11 energy channels between 0.14 and 6 MeV, the final fluxes being re‐calculated using a sophisticated fitting procedure after subtraction of proton counts (Morley et al., 2016). We use GPS omnidirectional electron flux averaged over 4.2 ≤ *L* ≤ 4.4, because it is measured at low geomagnetic latitudes <25° (as for Van Allen Probes data) where the flux is usually higher (Li et al., 2014; Thorne et al., 2013).

Morley et al. (2016) have noted that GPS 2‐MeV electron fluxes were usually ∼2 times smaller than 2.1‐MeV electron fluxes measured by the Van Allen Probes at the same *L*‐shells. We further checked that during the eight 10‐day periods following *Int*(*aa*_*H*_) events in March‐July 2013, the GPS 2‐MeV electron flux measured at *L* ∼ 4.2–4.4 was on average ∼2.5 times smaller than the 2.1 MeV flux simultaneously recorded at L* ∼ 4.5 by the Van Allen Probes (with identical average and median values of the 10‐day‐averaged Van Allen Probes to GPS flux ratio and 75% of these ratios within [1.6, 3.0]). This is probably mainly due to different calibrations of the detectors (Morley et al., 2016). Indeed, the GPS and Van Allen Probes measurements used here have been performed during quiet to moderately disturbed 10‐day periods beginning at the end of *Int*(*aa*_*H*_) events of strong geomagnetic activity—that is, such 10‐day periods usually start in the late recovery phase of weak to strong storms. At such nearly quiet times, the geomagnetic field usually comes back to a nearly dipolar configuration, such that L* ∼ 4.5 ∼ *L*. Therefore, the analyzed GPS and Van Allen Probes data are likely obtained at very similar radial distances from the Earth. The 10‐day time‐integrated fluxes obtained from GPS satellites at *L* ∼ 4.2–4.4 during these particular periods should also be weakly dependent on the exact magnetic field model used. The remarkable coherence of ∼1–2 MeV electron fluxes over the region 4.0 < *L* < 5.5 (with typical correlation lengths Δ*L* ∼ 0.5) after enhancement events (Pinto, Bortnik, et al., 2020; Walton et al., 2021) further confirms that the analyzed GPS fluxes obtained at *L* = 4.2–4.4 can be used as good proxies for fluxes at slightly higher *L*‐shells.

Accordingly, we select the ∼30 strongest (among a cluster, or isolated) *Int*(*aa*_*H*_) > 1,400 nT⋅hr events in 2003–2005 with available GPS electron flux and similarly the ∼60 strongest *Int*(*aa*_*H*_) events in 2012–2017. We multiply the GPS electron flux at *L* = 4.2–4.4 by a constant factor of 2.5 to obtain electron fluxes approximately equivalent to 2.1‐MeV electron fluxes measured by the Van Allen Probes at L* ∼ 4.5 during these quiet to weakly disturbed periods. To examine the effects of such strong *Int*(*aa*_*H*_) events on time‐integrated electron flux, we integrate electron flux over fixed 10‐day periods. The starting time *t*_0_ of such 10‐day periods is fixed at the end of each *Int*(*aa*_*H*_) event, or else at its starting time plus 3–4 days when a longer‐than‐3‐day *Int*(*aa*_*H*_) event has then already reached *Int*(*aa*_*H*_) > 1,400 nT⋅hr before its end (some examples of *Int*(*aa*_*H*_) events are provided in Appendix A). Indeed, 2–3 days of continuously elevated geomagnetic activity are usually sufficient to produce significant increases of 2‐MeV electron flux at L* ∼ 4.5, via a prolonged local acceleration by chorus waves and/or inward radial diffusion by ultra low frequency (ULF) waves (Baker et al., 1994; Horne et al., 2005; Mourenas, Artemyev, & Zhang, 2019; Ozeke et al., 2020; Thorne et al., 2013; Zhao et al., 2019).

**Figure 1:**
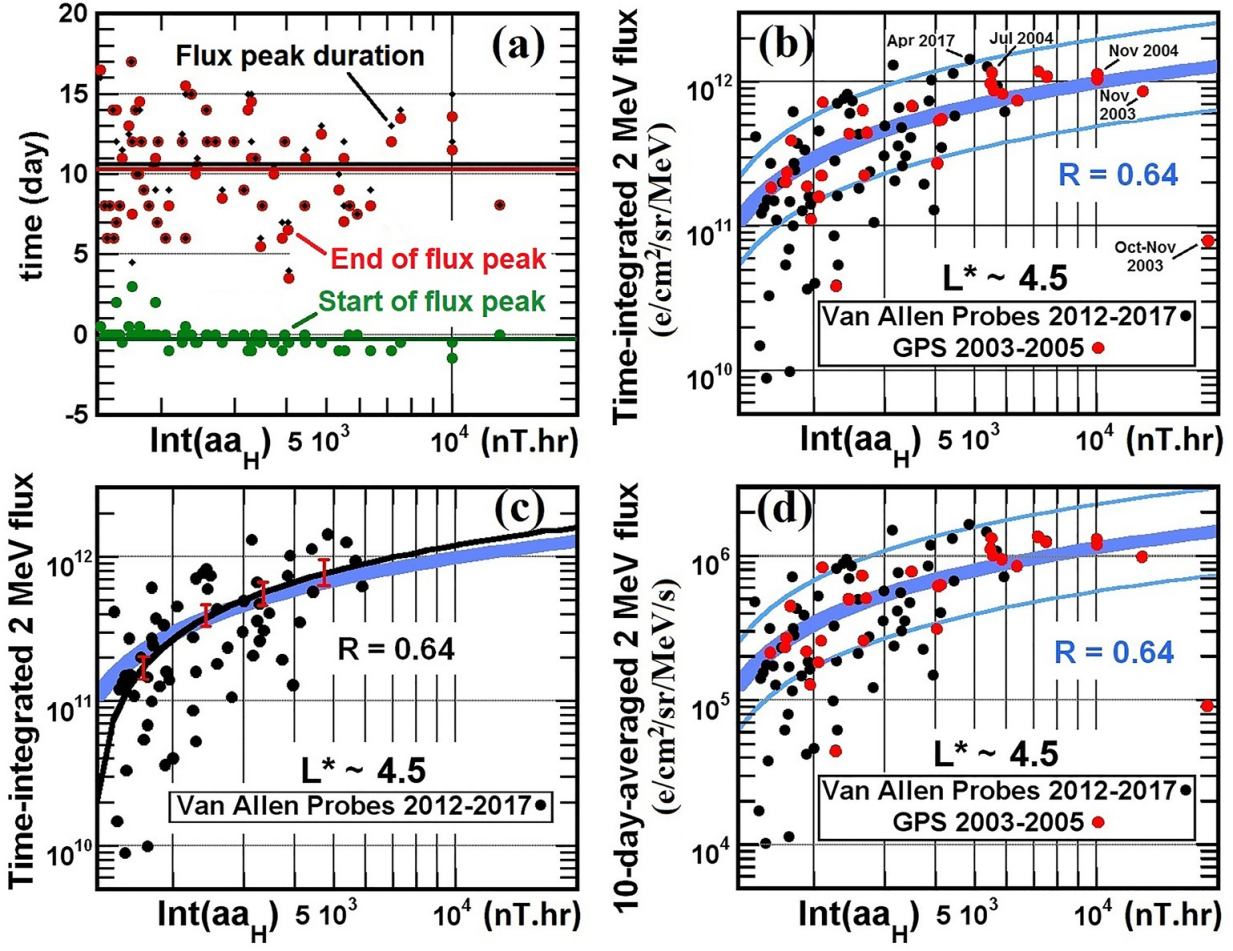
(a) Distribution of starting time (green), end time (red), and duration (black) of periods of high time‐integrated 2‐MeV electron flux measured by the Van Allen Probes near L* ∼ 4.5 or by GPS satellites at *L* = 4.2–4.4 following *Int*(*aa*_*H*_) > 1,400 nT⋅hr events. Time 0 corresponds to the end of an *Int*(*aa*_*H*_) event, or to its starting time plus 3–4 days if it is longer than 3 days and has already reached 1,400 nT⋅hr. Average values are shown by horizontal lines of same colors. (b) Time‐integrated 2‐MeV electron flux measured by the Van Allen Probes at L* ∼ 4.5 in 2012–2017 (black circles) and by GPS satellites at *L* = 4.2–4.4 (rescaled by a factor 2.5) in 2003–2005 (red circles) during 10‐day periods immediately following *Int*(*aa*_*H*_) > 1,400 nT⋅hr events, as a function of *Int*(*aa*_*H*_). The best least‐squares fit is shown by a thick blue curve. A factor 2 range around this best fit is delimited by thin blue curves. The dates of several events are indicated. (c) Same as (b) but keeping only time‐integrated electron fluxes measured by the Van Allen Probes in 2012–2017 (black circles). The corresponding best fit is shown in black (with standard error bars in red), together with the best fit from panel (b) in blue. (d) Same as (b) but showing the corresponding average electron flux over the same 10‐day periods.

To verify that using such fixed 10‐day periods of flux integration is appropriate for characterizing the impact of *Int*(*aa*_*H*_) events on space weather, we show in Figure 1a the distribution of the start and end times of the periods of high 2‐MeV electron flux measured by the Van Allen Probes or GPS satellites following the selected strong *Int*(*aa*_*H*_) > 1,400 nT⋅hr events. Such start and end times are respectively fixed at ∼1/2 and ∼1/3 of the maximum daily flux reached during each 10‐day period. Such start and end times of high flux periods are calculated here with respect to the time *t*_0_ defined above, corresponding to the end of each *Int*(*aa*_*H*_) event. We also indicate the full duration (between their start and end times) of these periods of high 2‐MeV electron flux. Figure 1a shows that *Int*(*aa*_*H*_) > 1,400 nT⋅hr events are followed by prolonged peaks of 2‐MeV electron flux, starting on average immediately at the time *t*_0_ and lasting in general 10 ± 4 days. The average start of such flux peaks occurs at *t* ≃ −0.15 days (slightly earlier than *t*_0_), their end at *t* ≃ 10.3 days, for a full duration of ≃10.5 days (with standard errors smaller than ±0.4 days). Accordingly, we henceforth examine time‐integrated electron fluxes calculated over these fixed 10‐day periods following strong *Int*(*aa*_*H*_) events.

The events with *Int*(*aa*_*H*_) > 1,400 nT⋅hr are very good precursors/predictors of prolonged peaks of 2‐MeV electron flux reaching 10‐day time‐integrated fluxes larger than 2 ⋅ 10^11^ e/cm^2^/sr/MeV, since they allow to recover nearly all (92%) of these flux peaks in 2012–2017. This is due to the fast decrease of the average time‐integrated flux as *Int*(*aa*_*H*_) decreases below 2000 nT⋅hr in Figure 1b. Therefore, decreasing the *Int*(*aa*_*H*_) threshold below 1,400 nT⋅hr would not significantly improve the modeling of these flux peaks. Conversely, increasing this threshold to 2,000 nT⋅hr would result in missing ∼30% of these events (see Figure 1b). This indicates that the 1,400 nT⋅hr threshold is appropriate.

Figure 1b shows that all *Int*(*aa*_*H*_) > 1,400 nT⋅hr events are followed by 10‐day periods of high time‐integrated 2‐MeV electron flux. There is a significant Pearson linear correlation *R* = 0.64 (*R* ^2^ = 0.41) between *Int*(*aa*_*H*_) and 10‐day‐integrated ∼2‐MeV electron fluxes *F*(*t*) at L* ∼ 4.5 measured by the Van Allen Probes or inferred from measurements by GPS satellites. The best least‐squares fit to the 10‐day‐integrated 2‐MeV electron flux is given by

(1) $$\int F\left(t\right)dt≃\left(0.4283\ln\left[Int\left(aa_{H}\right)\right]-2.963\right)\cdot 10^{12}\;\;{e/cm}^{2}/sr/MeV.$$

Nearly 81% (70%) of the measured values remain within a factor 2.5 (2) of the best fit in Equation 1 and ∼98% (95%) remain below 2.5 (2) times the best fit level. The Spearman Rank Order Correlation Coefficient (ROCC) between the obtained best least‐squares fit and the measured time‐integrated flux is ROCC = 0.67. The *z*‐score of the ROCC is *z* > 4, that is, the Fisher transformation of the ROCC is more than four standard deviations away (corresponding to a *p*‐value < 0.0001) from the null hypothesis of a statistical independence of time‐integrated flux and *Int*(*aa*_*H*_) in Figure 1b (e.g., Fieller et al., 1957). These results suggest that the dependence of time‐integrated flux on *Int*(*aa*_*H*_) is both real and statistically significant.

Figure 1c shows that the best fit to all Van Allen Probes and GPS data (in blue) given by Equation 1 is very close (within standard error bars) to the best fit to Van Allen Probes data alone (in black, corresponding to *∫F*(*t*)*dt* = (0.575 ln[*Int*(*aa*_*H*_)] − 4.10)10^12^ e/cm^2^/sr/MeV and *R* = 0.64). The difference between these two fits remains smaller than 20% between 1800 and 20,000 nT⋅hr, despite the absence of very strong *Int*(*aa*_*H*_) > 6,000 nT⋅hr events during the 2012–2017 interval, characterized by a weaker geomagnetic activity than in 2003–2005. This demonstrates that the fit to 2003–2005 and 2012–2017 data given in Equation 1 remains reliable even over a subset of the total time interval, where only data from the same satellite are used. Figures 1b and 1c also show that during these two very different solar cycles, an *Int*(*aa*_*H*_) event of a given strength produces on average the same 10‐day‐integrated electron flux. Therefore, the parameterization by *Int*(*aa*_*H*_) obtained in Figure 1b can be used with a reasonable confidence to estimate time‐integrated relativistic electron fluxes during other solar cycles.

Figure 1d finally shows that strong *Int*(*aa*_*H*_) events produce both high 10‐day fluences (as seen in Figure 1b) and high time‐averaged 2‐MeV electron fluxes, often exceeding 10^6^ e/cm^2^/sr/MeV/s. After rescaling, the same best least‐squares fit as in Figure 1b indeed provides a best fit to the time‐averaged 2‐MeV flux 〈*F*〉, as

(2) $$\langle F\rangle ≃\left(0.49566\ln\left[Int\left(aa_{H}\right)\right]-3.429\right)\cdot 10^{6}\;\;{e/cm}^{2}/sr/MeV/s.$$

It is worth emphasizing the logarithmic increase of the high time‐averaged relativistic electron fluxes found in Figure 1b, especially for *Int*(*aa*_*H*_) > 4,000 nT⋅hr. This may be due to the fact that the strongest *Int*(*aa*_*H*_) events often result from the impact on the magnetosphere of a long succession of solar wind structures (Tsurutani et al., 2006). Such a succession of solar wind disturbances generally leads to an initial dropout of relativistic electron flux, followed by a strong increase due to local chorus‐driven electron acceleration and/or inward radial diffusion of electrons by ULF waves (Mourenas, Artemyev, & Zhang, 2019; Murphy et al., 2018; Ozeke et al., 2020; Thorne et al., 2013; Tsurutani et al., 2006). However, it can ultimately lead to a second dropout of electron flux in the few days following the *Int*(*aa*_*H*_) event, thereby efficiently limiting the maximum 10‐day‐integrated flux. Many events of comparatively smaller time‐integrated flux in Figure 1b are probably partly due to such dropouts, produced by magnetopause shadowing, outward radial diffusion, or wave‐induced electron precipitation (Boynton et al., 2017; Mourenas et al., 2016; Olifer et al., 2018; Pinto, Zhang, et al., 2020; Shprits et al., 2006; Su et al., 2016) in the days following the *Int*(*aa*_*H*_) period. It is exactly what happened just after the huge *Int*(*aa*_*H*_) = 18,800 nT⋅hr Halloween superstorm of October 2003, when a subsequent solar flare impacted the magnetosphere on 4 November and led to a fast dropout, abruptly ending a period of enhanced electron flux only a few days after its start (Mourenas, Artemyev, & Zhang, 2019), resulting in an unexpectedly low 10‐day‐integrated flux level (indicated in Figure 1b).

### 2.3 Quantification of Model Performance

**Figure 2:**
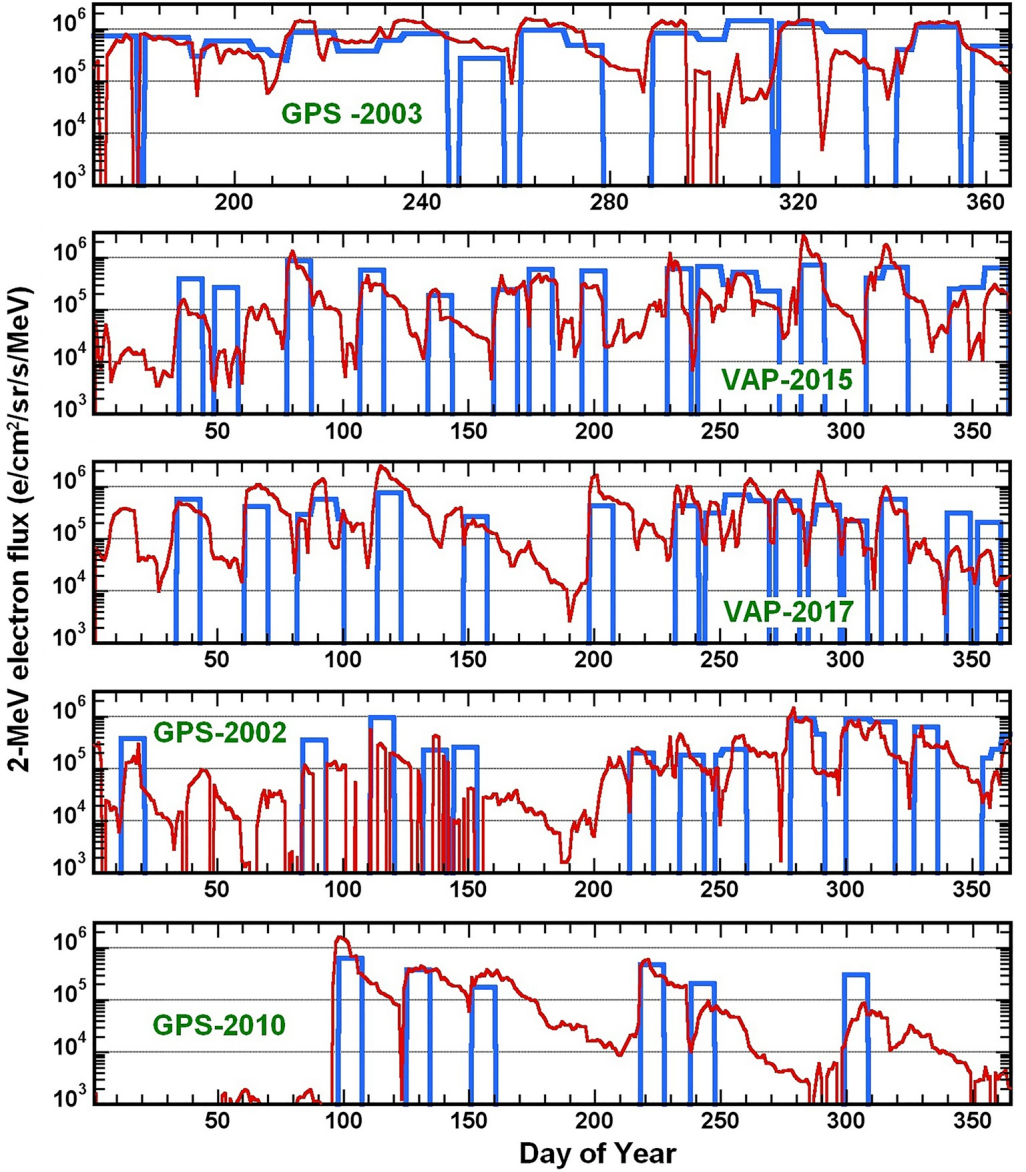
Daily‐averaged 2‐MeV electron flux (in e/cm^2^/sr/s/MeV) measured by the Van Allen Probes at L* ≃ 4.5 in 2015 and 2017 and by Global Positioning System satellites at *L* = 4.2 − 4.4 (rescaled by a factor 2.5) in 2002, 2003, 2010 (red), together with daily‐averaged 2‐MeV electron flux predicted by the model in Equation 2 after strong *Int*(*aa*_*H*_) > 1,400 nT⋅hr events (blue). Note that 2002 and 2010 are out‐of‐sample years.

To take into account the presence of successive *Int*(*aa*_*H*_) > 1,400 nT⋅hr events occurring in clusters during very active periods, we slightly refine our forecast model by simply assuming that the time‐averaged flux at a given time is equal to the maximum flux given by the best fit in Equation 2 based on the strongest *Int*(*aa*_*H*_) > 1,400 nT⋅hr event ending during the preceding 10 days. Comparisons between the modeled (or predicted) and measured 2‐MeV electron fluxes are displayed in Figure 2 during various years. Henceforth we use daily‐averaged 2‐MeV electron fluxes from GPS satellites at *L* ≃ 4.2–4.4 in 2001–2011 (multiplied by a factor 2.5 as before) and from the Van Allen Probes near L* ∼ 4.5 in 2012–2017. Qualitatively, Figure 2 shows that strong *Int*(*aa*_*H*_) > 1,400 nT⋅hr events are good predictors (and precursors) of long‐duration high 2‐MeV electron flux peaks, which correspond to periods of high time‐integrated fluxes. A similarly good qualitative agreement between model and data is found for the in‐sample years 2003, 2015, and 2017 used to build the model, and for the out‐of‐sample years 2002 and 2010 (near solar maximum and solar minimum, respectively), demonstrating the predictive capacity of the model. Nearly all strong *Int*(*aa*_*H*_) > 1,400 nT⋅hr events are indeed followed by periods of high time‐integrated 2‐MeV electron flux, while only a few of the highest long‐duration flux peaks are not predicted.

Several points are worth emphasizing concerning the training and validation procedures used for our forecast model. First, this forecast model mainly aims at predicting the 10‐day periods of highest time‐integrated electron flux in a distant future, presumably during a different solar cycle. To have a sufficient amount of meaningful data in the training set used for building the model, the years 2003–2005, containing some of the highest relativistic electron fluxes recorded in the past 20 years, have been selected in Section 2.2, together with 2012–2017 during which Van Allen Probes data is available. Second, to provide a fair test of the forecasting ability of a model in a distant future, the validation data set should be as independent as possible of the training data set (e.g., Wang et al., 2020). The existence of significant temporal correlations in the *aa* index time series (similar to *aa*_*H*_), corresponding to the 27‐day recurrence period of solar activity and its multiples up to 108 days (Lockwood et al., 2019), and the well‐known seasonal and yearly recurrences of geomagnetic storm patterns (Chapman, McIntosh, et al., 2020; Hathaway, 2015; Owens et al., 2021), suggest to use well‐separated groups of years for the validation data set and the training data set, to exclude possible correlations between these two data sets. This led us to select for the validation data set the out‐of‐sample years 2001–2002 and 2006–2011, which are sufficiently distant from the in‐sample years 2003–2005 and 2012–2017 of the training set. This way, moreover, a large majority (∼2/3) of the validation data set belongs to solar cycle 23, whereas a large majority of the training data set belongs to solar cycle 24. This should ensure that our validation data set is as independent as possible of our training data set, and representative of a distant period belonging to a different solar cycle. This validation data set will represent a hard test for the forecast model, but it should better show its skill and possible limitations related to the evolution of solar activity over successive cycles. Alternatively, we could have selected training and validation data sets more uniformly distributed over 2001–2017, by selecting for example, odd and even years. But this would have introduced some correlations between nearby years from the two sets, and the validation set would not anymore have been mainly selected during a different solar cycle, making it less representative of an independent period located in a distant future.

Hereafter, several quantitative measures of the model performance are provided. To quantify the performance of the model on all days of each year, we first provide in Table 1 the True Skill Statistics (TSS), Heidke Skill Score (HSS), probability of detection (POD), probability of false detection (POFD), and false alarm ratio (FAR) (Woodcock, 1976; Zheng et al., 2019) of the *Int*(*aa*_*H*_) model for the prediction of days of high daily‐averaged 2‐MeV electron flux higher than 3.5 ⋅ 10^5^ e/cm^2^/sr/MeV/s at L* ∼ 4.5. Such high daily fluxes have been observed only ∼15% of the time in 2001–2017. The POD (also known as hit rate) is equal to the fraction of actual events (here, days of high flux) correctly predicted, and the POFD is the fraction of non‐event days that were incorrectly forecast as events (Zheng et al., 2019). The FAR is the fraction of predicted events that turn out to be non‐events. The HSS is an equitable measure of categorical performance, based on the fraction of correctly predicted events after elimination of correct predictions arising purely from random chance (Heidke, 1926; Hogan & Mason, 2011). A null or negative HSS indicates no prediction skill, while a perfect model has a HSS of 1. The TSS (also known as Peirce Skill Score or Hanssen and Kuipers discriminant) is an equitable measure of categorical forecast performance similar to the HSS, based on the fraction of correctly predicted events after elimination of correct predictions arising from random chance, written as TSS = POD − POFD (Peirce, 1884; Woodcock, 1976). The TSS has the advantage of being unbiased with respect to event/non‐event sample ratio, allowing more accurate comparisons between skill scores for different samples than the HSS (Woodcock, 1976). A forecast model is deemed excellent for TSS ∈ (0.8, 1.0), good for TSS ∈ (0.6, 0.8), fair for TSS ∈ (0.4, 0.6), whereas TSS <0.2 indicates no predictive ability (Landis & Koch, 1977). However, the TSS may become less suitable than the HSS in the case of rare events with occurrences smaller than 1% (Doswell III et al., 1990).

**Table 1:** Skill Scores and Accuracy Metrics of the Model Given by Equation 2 Together With a Threshold *Int*(*aa*_*H*_) > 1,400 nT⋅hr for Predicting Individual Days of Average 2‐MeV Electron Flux 〈*F*〉 > 3.5 ⋅ 10^5^ e/cm^2^/sr/MeV/s, and of the Model Given by Equation 1 Together With *Int*(*aa*_*H*_) > 3,650 nT⋅hr for Predicting 10‐Day Periods With *∫F dt* > 8 ⋅ 10^11^ e/cm^2^/sr/MeV, Near L* ∼ 4.5 in 2001–2017

	2003–2005 and 2012–2017 (in‐sample)	2001–2002 and 2006–2011 (out‐of‐sample)	2001–2017 (ALL)
Skill and accuracy of model (2) with *Int*(*aa*_*H*_) > 1,400 nT⋅hr for predicting days of 〈*F*〉 > 3.5 ⋅ 10^5^ e/cm^2^/sr/MeV/s			
TSS	0.60	0.60	0.62
HSS	0.58	0.43	0.55
POD	0.73	0.68	0.72
POFD	0.13	0.08	0.10
FAR	0.35	0.64	0.43
MEF	1.6	3.1	2.0
SSPB	−30%	+210%	+43%
Skill and accuracy of model (1) with *Int*(*aa*_*H*_) > 3,650 nT⋅hr for predicting 10‐day periods with *∫F dt* > 8 ⋅ 10^11^ e/cm^2^/sr/MeV			
TSS	0.78	0.96	0.81
HSS	0.61	0.42	0.56
POD	0.84	1.0	0.86
POFD	0.06	0.04	0.05
FAR	0.48	0.7	0.55
MEF	1.4	2.1	1.6
SSPB	−15%	+110%	−10%

*Note.* The predicted 10‐day periods begin immediately after these *Int*(*aa*_*H*_) events, or at most 3–4 days after the start of longer‐than‐3‐day *Int*(*aa*_*H*_) events when *Int*(*aa*_*H*_) has already reached 1,400 or 3,650 nT⋅hr.

To quantify the accuracy of the model during days belonging to the predicted 10‐day periods of high flux following *Int*(*aa*_*H*_) > 1,400 nT⋅hr events, we use two additional metrics. The Median Error Factor (MEF) between predicted and measured values is derived from the Median Symmetric Accuracy (MSA) introduced by Morley et al. (2018), under the form MEF = 1 + MSA/100 = exp(*M*(| ln(*Q*_*i*_)|)), where *M* denotes the median and *Q*_*i*_ the ratio of modeled to measured values (Glauert et al., 2018; Morley et al., 2018). Half of the predictions remain within a factor of MEF of the data. The MEF and MSA metrics are especially appropriate for electron flux data spanning several orders of magnitude, and robust to the presence of outliers or bad data (Morley et al., 2018; Zheng et al., 2019). In Table 1, the MEF is first calculated for daily time‐averaged 2‐MeV electron fluxes during the 10‐day periods predicted by the *Int*(*aa*_*H*_) model using Equation 2. For this evaluation, the predicted continuous 10‐day periods of high time‐integrated flux are therefore split into 10 separate days. This procedure has the advantage of increasing the number of points, but at the expense of somewhat underestimating the true skill of the model in predicting long‐duration periods of high time‐integrated flux, since the accuracy of such predictions only requires a good performance on the *n* < 10 days of highest flux within each 10‐day period. The symmetric signed percentage bias (SSPB), defined as SSPB = 100 *Sgn*(*M*(ln(*Q*_*i*_)))(exp(|*M*(ln(*Q*_*i*_))|) − 1) (with *Sgn* the sign function), gives a robust and unbiased measure of the mean percentage error of the model (Morley et al., 2018).

An intrinsic limitation of the present model is that it cannot forecast low to moderate electron fluxes occurring outside of the predicted 10‐day periods of high flux. But this should not be considered a problem in practice, since this type of model is mainly designed to provide advance warning of periods at risk of high time‐integrated flux and TID.

The high probability of detection POD(days) ≃ 0.68 − 0.72 found in Table 1 shows that a large majority (∼70%) of the days of high 2‐MeV electron flux are correctly predicted, during both in‐sample years (used to derive the model) and out‐of‐sample years. Note also that part of the non‐predicted days of high flux simply belong to periods of high flux lasting longer than the assumed 10 days. The POFD is much smaller than the POD, with POFD(days) ≃ 0.1. This gives a True Skill Statistics TSS(days) ≃ 0.62, indicating a good forecast efficiency (Landis & Koch, 1977) for both the in‐sample and out‐of‐sample data sets. The HSS(days) ≃ 0.43 − 0.55 is similarly elevated. It is worth emphasizing that the high TSS and HSS skill scores of the model correspond to days of high flux that are predicted, on average, 5 days in advance (between 0 and 10 days ahead).

The Median Error Factor is MEF(days) ≃ 2 for the full data set, showing a good accuracy for electron fluxes that vary by orders of magnitude (similar to the accuracy of much more sophisticated models; e.g., see Glauert et al., 2018). The MEF is larger (∼3) during out‐of‐sample years than during in‐sample years (∼1.6). These different MEF values could stem from the different origins of the data at L* ∼ 4.5, since 63% of the in‐sample data directly come from Van Allen Probe measurements at L* ∼ 4.5, whereas 100% of the out‐of‐sample data are inferred from measurements by GPS satellites at *L* = 4.2–4.4, slightly closer to the Earth, which introduces some additional uncertainty. Alternatively, these different MEF values could be due to the fact that 75% of the out‐of‐sample years (i.e., 2006–2011) take place around solar minimum, whereas all in‐sample years belong to solar maximum or the declining phase of a solar cycle. The periods of solar maximum and declining phase are known to be more active in terms of geomagnetic storms and substorms (driven by coronal mass ejections or high‐speed solar wind streams) than during solar minimum (Richardson et al., 2000; Tsubouchi & Omura, 2007; Tsurutani et al., 2006). The weakest storms present during solar minimum are expected to produce enhancements of 2‐MeV electron flux and phase space density (PSD) at higher *L* than stronger storms (Tverskaya et al., 2003; Zhao & Li, 2013). The subsequent enhancement of 2‐MeV electron flux at L* ∼ 4.5 may then occur after a significant delay, because these electrons need to be diffused radially inward to L* ∼ 4.5 by ULF waves from their initial PSD peak at L* ≃ 5–6 (Li et al., 2014). This could explain the higher MEF, FAR and SSPB and the slightly lower HSS during out‐of‐sample years. But we checked that only a minor fraction of the days of false alarm (erroneously predicted) during out‐of‐sample years indeed correspond to prolonged peaks of 2‐MeV electron flux starting sensibly later than their predicted start (like in September and November 2010 in Figure 2), and that most solar minimum flux peaks are as well predicted (like in April‐August 2010 in Figure 2) as solar maximum flux peaks in 2013–2017. Actually, the majority of the days of false alarm (erroneously predicted) belong to 2001 and the first half of 2002, near solar maximum. This particular period corresponds to more frequent large geomagnetic storms (reaching *Dst* < −130 nT) than in 2003–2004 or 2013–2015 (near the next solar maximum). Such large storms, which are mostly caused by coronal mass ejections, are usually less efficient than weaker storms caused by corotating interaction regions and high‐speed solar wind streams in producing prolonged peaks of 2‐MeV electron flux at *L* > 4 in the outer belt (Miyoshi & Kataoka, 2011; Mourenas, Artemyev, & Zhang, 2019; Spasojevic, 2014). Nevertheless, it is worth noting that the SSPB of the model over the full data set is limited, with SSPB ∼+43%, indicating daily predicted fluxes only slightly higher than measured fluxes.

The results in Figure 1b also suggest a model for predicting the 10‐day periods of highest time‐integrated 2‐MeV electron flux *∫F dt* > 8 ⋅ 10^11^ e/cm^2^/sr/MeV near L* ∼ 4.5, given by Equation 1 together with a threshold *Int*(*aa*_*H*_) > 3,650 nT⋅hr. Such 10‐day periods of high time‐integrated measured electron flux have been observed only ∼4% of the time in 2001–2017, all of them after *Int*(*aa*_*H*_) > 1,900 nT⋅hr events (and 63% after *Int*(*aa*_*H*_) > 5,000 nT⋅hr events). Such 10‐day periods are the most dangerous time intervals for satellites in terms of Total Ionizing Dose. Table 1 shows that both the TSS and HSS of this forecast model are high, with TSS = 0.81 and HSS = 0.56 (and POD = 0.86) over the full 2001–2017 data set, indicating its good to excellent efficiency. The TSS is even higher (0.96) during out‐of‐sample years, although the HSS is reduced to 0.42. This is simply due to the ∼4 times more rare occurrences of such 10‐day periods of high time‐integrated measured flux during out‐of‐sample years: although this leads to a higher FAR, it is important to note that all real events are correctly predicted (POD = 1) during out‐of‐sample years, despite the fact that they are observed during solar minimum. Moreover, during both out‐of‐sample and in‐sample years, roughly 65% of the false alarms still correspond to 10‐day periods of high time‐integrated flux *∫F dt* > 2.66 ⋅ 10^11^ e/cm^2^/sr/MeV that represent an important risk. The moderate values of MEF ≃ 1.6 and 2.1 and SSPB ≃ −10% and +110% over the full data set and over out‐of‐sample years, respectively, show that the accuracy of this model is significantly higher than for predicting individual days of high flux. Increasing the threshold to *Int*(*aa*_*H*_) > 5,000 nT⋅hr in the model still gives high TSS ≃ HSS ≃ 0.6 over 2001–2017, but lower POD ≃ 0.63 and FAR ≃ 0.4. Despite the intrinsic limitations of these models, we underline that most of the days of high time‐integrated flux inside the predicted 10‐day periods are predicted more than 2–3 days in advance, based on the past history of only one ground‐based geomagnetic index—without any needed input from satellites, contrary to many other forecast models (e.g., Boynton, Balikhin, et al., 2016; Chu et al., 2021; Glauert et al., 2021; Pires de Lima et al., 2020).

### 2.4 Physical Insights

How to explain the formation and duration of the long 10‐day periods of high time‐integrated relativistic electron flux analyzed in Section 2.1? Let us first examine their formation at L* ∼ 4.5. Whistler‐mode chorus wave intensity is correlated with geomagnetic activity (Agapitov et al., 2015, 2018; Li et al., 2009; Meredith et al., 2003). Therefore, disturbed periods of high *Int*(*aa*_*H*_) can lead to a local quasi‐linear energization of part of the abundant 100–300 keV electrons up to ∼2 MeV via cyclotron resonance with intense ($B_{w}^{2}∼100^{2}$ pT^2^) chorus waves, producing elevated fluxes of 2‐MeV electrons over time scales of ∼1–3 days in the low density region near L* ∼ 4.5 when *AE* ∼ 400–600 nT (O. Agapitov et al., 2019; Horne et al., 2005; Thorne et al., 2013; Summers et al., 1998), roughly corresponding to an average *aa*_*H*_ ∼ 50–100 nT (Rostoker, 1991). Including non‐linear interactions with a population of more intense, but relatively short and phase‐decorrelated, chorus wave packets should produce an only moderately faster electron acceleration over 1–3 days (Artemyev et al., 2021; Zhang et al., 2020). ULF wave intensity is also correlated with geomagnetic activity (Ozeke et al., 2014). Accordingly, a prolonged inward radial diffusion of electrons by ULF waves during disturbed *Int*(*aa*_*H*_) periods can also explain the formation of elevated 2‐MeV electron fluxes near L* ∼ 4.5 over time scales of 1–3 days for *Kp* ∼ 4–5 (Ozeke et al., 2014, 2020), roughly corresponding to an average *aa*_*H*_ ∼ 50–100 nT. Chorus and ULF wave driven electron acceleration can occur separately, simultaneously, or in close succession (Li et al., 2014).

**Figure 3:**
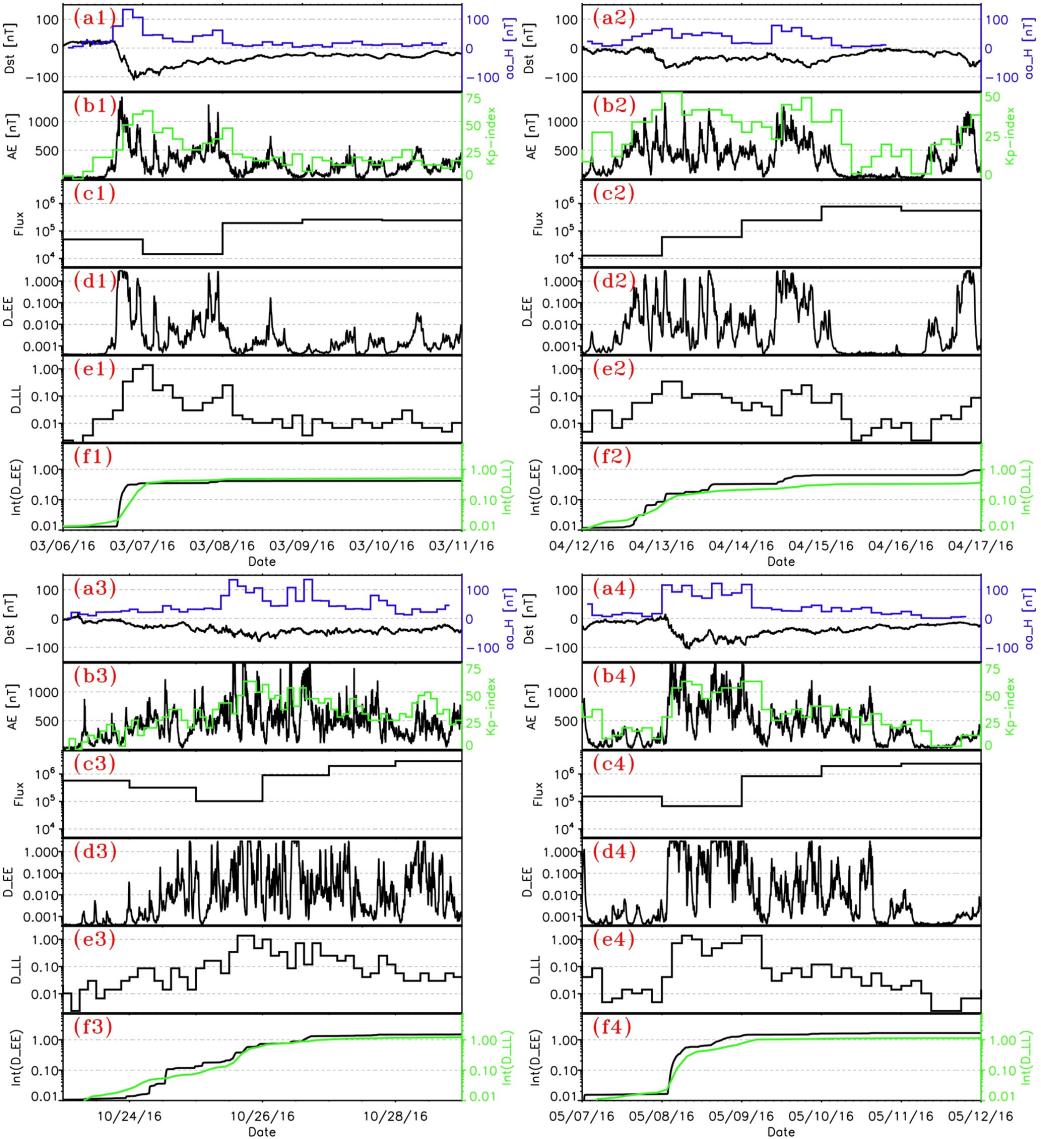
(a1–a4) *aa*_*H*_ (blue) and *Dst* (black) indices during four events in 2016 (top: moderate *Int*(*aa*_*H*_) events, bottom: strong *Int*(*aa*_*H*_) events). (b1–b4) *AE* (black) and *Kp* × 10 (green). (c1–c4) Daily‐averaged 2‐MeV electron flux (in e/cm^2^/sr/s/MeV) measured by the Van Allen Probes at L* ≃ 4.5. (d1–d4) Chorus wave‐driven quasi‐linear energy diffusion rate *D*_*EE*_ at *L* = 4.5. (e1–e4) ULF wave‐driven radial diffusion rate *D*_*LL*_ at *L* = 4.5. (f1–f4) Time‐integrated *Int*(*D*_*EE*_) (black) and *Int*(*D*_*LL*_) (green) during these intervals.

To investigate these potential sources of 2‐MeV electrons, we use analytical estimates of the MLT‐averaged and bounce‐averaged quasi‐linear energy diffusion rate *D*_*EE*_(*AE*) of 1‐MeV electrons by chorus waves at *L* = 4.5 (Mourenas et al., 2014), provided by O. Agapitov et al. (2019) based on Van Allen Probes statistics of simultaneously measured local chorus wave frequency, amplitude, and plasma density. We show such diffusion rates *D*_*EE*_(*AE*) in Figure 3 during four time intervals corresponding to two moderate and two strong *Int*(*aa*_*H*_) events. The analytical electric field radial diffusion rate *D*_*LL*_(*Kp*) of electrons by ULF waves at *L* = 4.5 from Ozeke et al. (2014) is also shown, together with the time‐integrated *D*_*EE*_ and *D*_*LL*_ during these events, denoted as *Int*(*D*_*EE*_) and *Int*(*D*_*LL*_).

The two moderate *Int*(*aa*_*H*_) ≃ 1,600–1,950 nT⋅hr events took place on 6–7 March and 12–13 April 2016, in the wake of strong (min(*Dst*) ∼ −100 nT) and moderate (min(*Dst*) ∼ −60 nT) geomagnetic storms. These two moderate *Int*(*aa*_*H*_) events of continuously high *aa*_*H*_ > 18 nT last only ∼1.5 days, mostly during the recovery phase of the first geomagnetic storm, corresponding to increases by factors of ∼5–20 of the 2‐MeV electron flux from its initial level (see Figure 3). However, on 14 April 2016, just after a first *Int*(*aa*_*H*_) ≃ 1,600 nT⋅hr event, a second, much weaker *Int*(*aa*_*H*_) = 950 nT⋅hr event occurred during a second moderate storm, corresponding to a second, weaker increase of 2‐MeV flux by a factor ∼2–3. The lowest flux reached during storm main phase is likely related to both the *Dst* effect of adiabatic electron motion (Kim & Chan, 1997) and magnetopause shadowing loss (Shprits et al., 2006; Turner et al., 2012). The final 2‐MeV electron flux is ∼(2.5 − 5.5) × 10^5^ e/cm^2^/sr/MeV/s after these two moderate events.

The two strong *Int*(*aa*_*H*_) ≃ 3,900 and 5,700 nT⋅hr events took place on 8–10 May, and 24–27 October 2016. They correspond to a strong (min(*Dst*) ∼ −95 nT) storm and a moderate (min(*Dst*) ∼ −65 nT) storm, respectively. These *Int*(*aa*_*H*_) events start during storm main phase and continue for ∼2 days during the recovery phase. They correspond to similar enhancements (by factors of ∼6–15) of the 2‐MeV electron flux from its initial level as during the two moderate *Int*(*aa*_*H*_) events. However, the final 2‐MeV electron flux is ∼(2.4 − 2.9) × 10^6^ e/cm^2^/sr/MeV/s, roughly ∼5–10 times higher than after the two moderate *Int*(*aa*_*H*_) events.

Therefore, the final 2‐MeV electron flux appears to depend on the strength of these *Int*(*aa*_*H*_) events, as in statistical results displayed in Figure 1. Since the initial 2‐MeV flux varies wildly from event to event, it suggests that the factor of increase of the 2‐MeV flux from its initial level is not the appropriate parameter to classify these events. Instead, it is important to note that the final 2‐MeV flux is usually deconnected from its initial level by a steep dropout of electron flux occurring during storm main phase (or slightly earlier) due to magnetopause shadowing (Murphy et al., 2018; Turner et al., 2014, 2012). This is analogous to a ’hard reset’ of the outer radiation belt (Turner et al., 2014, 2012). In such a case, the final 2‐MeV flux level should be determined only by the time‐integrated strength of the chorus‐driven energization rate *D*_*EE*_ and of the radial diffusion rate *D*_*LL*_ of electrons, and by the flux level of 100–300 keV seed electrons injected from the plasma sheet that are accelerated to 2‐MeV (Horne et al., 2005; Ozeke et al., 2020). But during storm main phase and recovery phase, the PSD of 100–300 keV injected electrons at L* ∼ 4–6 remains nearly identical (within a factor of ∼2) from event to event in 2012–2016 statistics (Murphy et al., 2018). Consequently, the final 2‐MeV electron flux should be mostly determined by the time‐integrated *D*_*EE*_ and *D*_*LL*_. This conjecture is supported by the results in Figure 3, which show that the time‐integrated *Int*(*D*_*EE*_) and *Int*(*D*_*LL*_) indeed reach higher levels during stronger *Int*(*aa*_*H*_) events and correspond to a higher final 2‐MeV electron flux. Electron energization related to *Int*(*D*_*EE*_) and *Int*(*D*_*LL*_) apparently starts within a few hours of the flux dropout, as soon as injected 100–300 keV electrons can be accelerated without being immediately lost (Murphy et al., 2018; Turner et al., 2012). The higher cumulative rates of chorus wave‐driven energization, *Int*(*D*_*EE*_), and inward radial diffusion, *Int*(*D*_*LL*_), of electrons during the stronger *Int*(*aa*_*H*_) events likely explain the formation of much higher fluxes of 2‐MeV electrons.

Finally, the average ∼10‐day duration of the prolonged periods of high 2‐MeV electron flux still needs to be explained. The dynamic evolution of the background plasma density *N*_*e*_ during and after a storm likely plays an important role in both the formation and duration of such 10‐day peaks of flux. Since the chorus wave‐driven electron acceleration rate *D*_*EE*_ varies like $1/N_{e}^{3/2}$, the reduction of plasma density due to plasmasphere erosion during the initial phase of a storm can strongly increase 2‐MeV electron flux near *L* = 4.5, just outside the plasmapause, rapidly forming such peaks of flux in one or a few days (Agapitov et al., 2019; Horne et al., 2005; Summers et al., 1998). After the storm, plasmasphere refilling takes place over a few more days, until the plasmapause usually reaches *L* > 4.5 again (Goldstein et al., 2014; O’Brien & Moldwin, 2003). Most of the 10 days of high flux occur during the following period, characterized by a weak geomagnetic activity, when the *L* ∼ 4.5 region should be either just above or inside the plasmasphere. But, both outside and inside the plasmasphere, the lifetimes of 2‐MeV electrons due to chorus or hiss wave‐driven precipitation into the atmosphere are statistically longer than 10 days during such moderately active periods (Agapitov et al., 2020; Aryan et al., 2020; Mourenas et al., 2017), that is, insufficiently short to explain the ending of a flux peak in less than ∼10 days.

However, if electromagnetic ion cyclotron (EMIC) waves are simultaneously present at *L* ∼ 4.5 inside high‐density regions where they can resonantly interact with not‐too‐high energy electrons (Summers et al., 1998), either within the refilled plasmasphere or inside a remnant plasmaspheric plume, a combined pitch‐angle scattering of 2‐MeV electrons by both whistler‐mode and EMIC waves (even at different local times) can reduce the lifetimes of 2‐MeV electrons to less than 10 days (Li et al., 2007; Mourenas et al., 2016; Pinto et al., 2019; Zhang et al., 2017). This has been confirmed by electron lifetime measurements around *L* = 4.5 during weakly disturbed periods with *Kp* ∼ 1–2 and 〈*AE*〉 < 200 nT (Mourenas et al., 2021, 2017).

An alternative explanation for the relatively short duration of these 10‐day periods of high flux could be the presence of a dropout of 2‐MeV electron flux caused by magnetopause shadowing and electron outward radial diffusion by ULF waves toward the last closed drift shell (Boynton et al., 2017; Olifer et al., 2018; Ozeke et al., 2020; Pinto, Zhang, et al., 2020; Shprits et al., 2006). Although such dropouts statistically occur only after a median waiting time of ∼20 days at *L* ∼ 4.2 (Boynton et al., 2017), the presence of a plasmaspheric plume during storm recovery (Goldstein et al., 2014) can facilitate the dropout by allowing an easier propagation of intense ULF waves to low *L* ∼ 4 (Degeling et al., 2018). The shorter duration of these flux peaks at higher *L* (Mourenas, Artemyev, & Zhang, 2019) is also consistent with the more frequent occurrence of dropouts at higher *L* (Boynton, Mourenas, & Balikhin, 2016; Boynton et al., 2017). Therefore, a dropout due to magnetopause shadowing or strong electron precipitation by combined EMIC and whistler‐mode waves, favored by the appearance of high density plasmaspheric regions at *L* ≥ 4.5 during the late recovery phase of a storm, can probably account for the average ∼10‐day duration of the analyzed periods of high time‐integrated 2‐MeV electron flux.

## 3 Distribution of *Int*(*aa*_*H*_) Events in 1868–2017

### 3.1 Distribution of Extreme *Int*(*aa*_*H*_) Events

In the homogenized *aa* index, called *aa*_*H*_, individual *aa* data points have been modified using time‐ and station‐dependent scale factors to correct *aa* for secular changes (Lockwood, Chambodut, Barnard, Owens, & Clarke, 2018; Lockwood, Chambodut, Barnard, Owens, Clarke, & Mendel, 2018). However, some information has been lost through the initial logarithmic discretization of the *aa* index and the existence of an upper limit *K* = 9 for the related *K* index (Chapman, Horne, & Watkins, 2020; Mayaud, 1980). Consequently, the maximum value of *aa*_*H*_ reached during an event does not quantify well the real extremum of activity, and Extreme Value Theory (Coles, 2001; Tsubouchi & Omura, 2007) is not directly applicable to individual *aa*_*H*_ values (Chapman, Horne, & Watkins, 2020). Nevertheless, Chapman, Horne, and Watkins (2020) have shown that yearlong averages of the largest 0.5% of *aa*_*H*_ values (corresponding to averages over 14–15 *aa*_*H*_ points) can still be used as reliable estimates of extreme activity. In a similar way, we use here as an estimate of extreme activity an integrated parameter, *Int*(*aa*_*H*_), equal to the sum of all *aa*_*H*_ values (corresponding to ∼10–30 *aa*_*H*_ points) recorded during an event where *aa*_*H*_ remains continuously above 18 nT. This summation over time, as long as *aa*_*H*_ remains above a low threshold, should partly correct for both the discretization of the *aa*_*H*_ index and the upper limit on *K* and *aa*_*H*_. Indeed, only a small number (at most one or two in general) of the *aa*_*H*_ values reach extremely high levels *aa*_*H*_ > 450 nT (or non‐homogenized *aa* > 450 nT) during a given *Int*(*aa*_*H*_) event. This means that more than ∼90% of the summed *aa*_*H*_ values during an *Int*(*aa*_*H*_) event correspond to a *K* index smaller than 8.2 (Mayaud, 1980) and should not be affected by the saturation *K* ≤ 9 of the corresponding *K* index. Accordingly, the potential error on the integrated quantity *Int*(*aa*_*H*_) should remain small. In addition, *Int*(*aa*_*H*_) clearly has no pre‐determined upper limit, since in principle it may increase indefinitely with the duration of integration of *aa*_*H*_ (i.e., with the duration of the event). This justifies the applicability of Extreme Value Theory for examining the probability of rare extreme *Int*(*aa*_*H*_) events (Coles, 2001).

In analogy to the central limit theorem, it has been shown that the exceedances over a threshold in a sample of *N* independent extreme events tend to follow a Generalized Pareto Distribution (GPD) for sufficiently high *N* and threshold values (Coles, 2001). A reliable Extreme Value Theory method therefore consists in fitting the tail of the distribution of exceedances of independent (by construction) *Int*(*aa*_*H*_) events over a well‐chosen and sufficiently high threshold min[*Int*(*aa*_*H*_)] by a GPD of the form

(3) $$P_{\mathrm{GPD}}\left(\xi ,\sigma \right)=\frac{1}{\sigma }{\left(1+\frac{\xi }{\sigma }\cdot \left(Int\left(aa_{H}\right)-\min\left[Int\left(aa_{H}\right)\right]\right)\right)}^{-1-1/\xi }$$

for its probability distribution (Coles, 2001).

**Figure 4:**
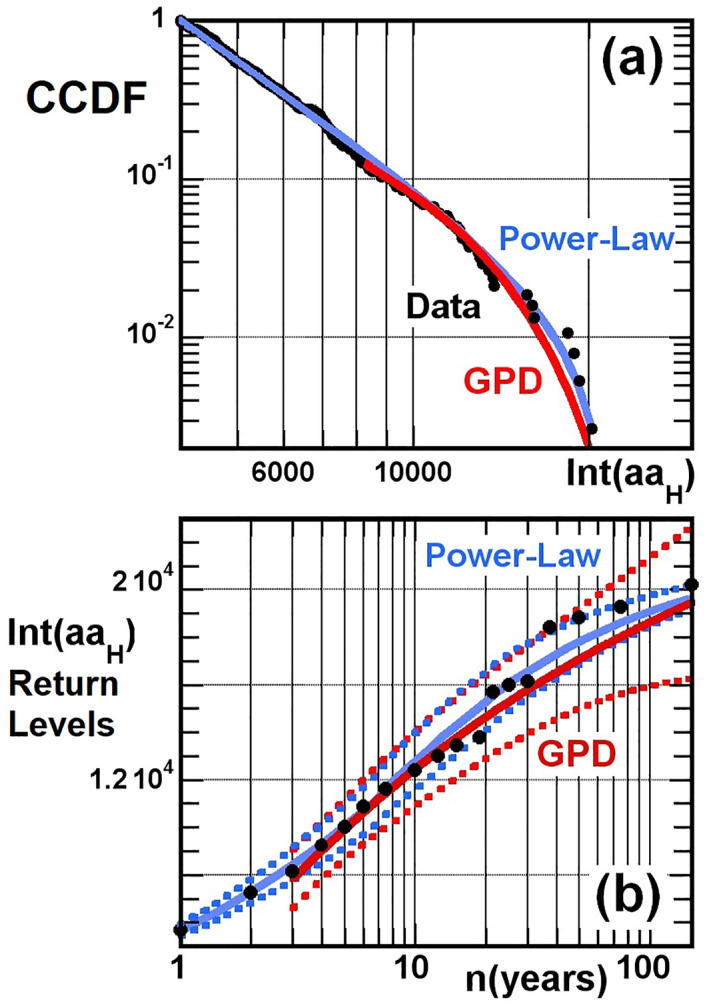
(a) Complementary to the Cumulative Distribution Function of *Int*(*aa*_*H*_) events (black circles), with corresponding best ML power‐law (with upper‐cutoff) fit (blue) and best ML Generalized Pareto Distribution (GPD) fit (red). (b) Return levels of observed *Int*(*aa*_*H*_) events (black circles) as a function of the period considered (in number of years), with GPD fit (red) and ML power‐law fit (blue). Dotted lines show 95% confidence intervals (for clarity, not all data points are shown at *Int*(*aa*_*H*_) < 13,750 nT⋅hr, all of them remaining within 95% confidence intervals).

The appropriate threshold min[*Int*(*aa*_*H*_)] for a reliable GPD fit corresponds to a sufficiently high range of min[*Int*(*aa*_*H*_)], where a linear relationship should exist between mean exceedance $\langle Int\left(aa_{H}\right)-\min\left[Int\left(aa_{H}\right)\right]\rangle$ and min[*Int*(*aa*_*H*_)], as well as nearly stable (constant) estimates of GPD parameters *ξ* and σ* = *σ* − *ξ* ⋅ min[*Int*(*aa*_*H*_)] obtained via the Maximum Likelihood (ML) method (Coles, 2001; Love et al., 2015; Tsubouchi & Omura, 2007). The optimal (*ξ*, *σ*) usually correspond to the lowest appropriate threshold min[*Int*(*aa*_*H*_)], because more events are taken into account (Coles, 2001). Here, it gives min[*Int*(*aa*_*H*_)] = 8,400 nT⋅hr (higher min[*Int*(*aa*_*H*_)] give similar *ξ* and *σ* but larger uncertainties, see details in Appendix B), *ξ* ≃ −0.213 ± 0.33, and *σ* ≃ 4,260 ± 1,880, where minimum and maximum parameter values correspond to 95% confidence intervals calculated via the delta method (Coles, 2001). Figure 4a shows the Complementary to the Cumulative Distribution Function (CCDF) of extreme *Int*(*aa*_*H*_) > 4,260 nT⋅hr events. The CCDF, also called “tail distribution”, gives the fraction of events with higher *Int*(*aa*_*H*_) than a given *Int*(*aa*_*H*_) value in abscissa. The GPD fit (red curve) is fairly close to the data (black points) in Figure 4a, with a maximum Kolmogorov‐Smirnov distance *D* ≃ 0.053 between the fitted CCDF and the data (Clauset et al., 2009), corresponding to a *p*‐value = 0.88. Accordingly, the hypothesis that extreme *Int*(*aa*_*H*_) events actually have such a GPD distribution cannot be confidently rejected at the *p* ≤ 0.05 level, and this GPD fit appears plausible (Coles, 2001). In addition, *ξ* remains negative over a 90% confidence interval, indicating the likely presence of an upper limit max[*Int*(*aa*_*H*_)] ≈ 20,000–28,000 nT⋅hr (a previous analysis of the *Int*(*aa*) data set has yielded similar results, see Mourenas, Artemyev, Zhang, et al., 2019).

An alternative ML fit to the CCDF of *Int*(*aa*_*H*_) is searched in the form of a power‐law yearly probability distribution with upper‐cutoff $P_{y}\left[Int\left(aa_{H}\right)\right]=C\cdot H\left(\max\left[Int\left(aa_{H}\right)\right]-Int\left(aa_{H}\right)\right)/Int{\left(aa_{H}\right)}^{\alpha }$ (with H the Heaviside function and an upper‐cutoff max[*Int*(*aa*_*H*_)] ≃ 21,000 nT⋅hr), as done before for time‐integrated *Dst* events (Mourenas et al., 2018). For a threshold *Int*(*aa*_*H*_) > 4,000 nT⋅hr (using lower thresholds yielded less good fits), we obtain *α* = 3.583 and *C* = 1.32 ⋅ 10^10^, with a small maximum Kolmogorov‐Smirnov distance *D* = 0.043 between fit and data, corresponding to a p‐value of 0.5 for 376 data points. Accordingly, the hypothesis that extreme *Int*(*aa*_*H*_) events have such a power‐law distribution with upper‐cutoff cannot be confidently rejected at the *p* ≤ 0.05 level (Coles, 2001). This ML power‐law fit performs well in Figure 4a (see blue curve). The root‐mean‐squared error (RMSE) between the power‐law fit and the CCDF of *Int*(*aa*_*H*_) > 4,000 nT⋅hr is small, RMSE = 0.016, with a squared Pearson correlation coefficient *R* ^2^ = 0.998, indicative of a very good fit. We also checked that this ML power‐law fit is only weakly sensitive to the choice of the upper‐limit max[*Int*(*aa*_*H*_)], since varying it by 5% changes CCDF values by less than 1% on average for the 10 highest *Int*(*aa*_*H*_) values, and much less at lower *Int*(*aa*_*H*_). The delta method applied to the ML estimate of *α* (Coles, 2001) gives a 95% confidence interval 3.26 < *α* < 3.9 (other methods give similar values, see Clauset et al., 2009). Therefore, this ML power‐law fit appears plausible, and it remains accurate over a much wider *Int*(*aa*_*H*_) domain than the GPD fit. Interestingly, the peak *aa*_*H*_ magnitude of *aa*_*H*_ > 40 nT storms has also an approximately power‐law distribution (Haines et al., 2019).

Physically, a power‐law distribution of the most extreme *Int*(*aa*_*H*_) events could result from protracted periods of strong solar wind driving that compel the magnetosphere‐ionosphere system to assume a particular self‐organized critical configuration in nearly stable non‐equilibrium (Aschwanden et al., 2016; Valdivia et al., 2013). A saturation process progressively more efficient from *Int*(*aa*_*H*_) ≈ 6,000 nT⋅hr to *Int*(*aa*_*H*_) = 20,000 nT⋅hr could also limit the decrease of the probability to get very strong events as *Int*(*aa*_*H*_) increases toward its upper limit max[*Int*(*aa*_*H*_)], potentially leading to an approximate power‐law shape (Mourenas et al., 2018; Mourenas, Artemyev, Zhang, et al., 2019; Zhang & Du, 2010). Such a saturation could stem from various physical mechanisms, such as enhanced ring current dropout due to magnetopause shadowing and outward radial diffusion (Boynton et al., 2017; Turner et al., 2014), ionospheric feedback (Toledo‐Redondo et al., 2021), or saturation of the solar wind‐magnetosphere coupling during stronger events (Kivelson & Ridley, 2008; Lopez et al., 2010; Siscoe et al., 2002).

### 3.2 Return Levels of Extreme *Int*(*aa*_*H*_) Events

For risk assessment, it is useful to consider the *n*‐year return level $Int{\left(aa_{H}\right)}_{n}$ of an extreme event—the expected *Int*(*aa*_*H*_) level exceeded once every *n* years. For extreme *Int*(*aa*_*H*_) ≥ min[*Int*(*aa*_*H*_)] = 8,400 nT⋅hr events in 1868–2017 having a GPD, it can be estimated as

(4) $$Int{\left(aa_{H}\right)}_{n}≃\min\left(Int\left(aa_{H}\right)\right)+\frac{\sigma }{\xi }\left[{\left(\frac{44n}{150}\right)}^{\xi }-1\right],$$

with *ξ* = −0.213 and *σ* = 4,260 (Coles, 2001). For the power‐law distribution ML fit with upper cutoff, it is given by

(5) $$Int{\left(aa_{H}\right)}_{n}={\left(\frac{150}{376n}\left[\min\;{\left(Int\left(aa_{H}\right)\right)}^{\beta }-\max\;{\left(Int\left(aa_{H}\right)\right)}^{\beta }\right]+\max\;{\left(Int\left(aa_{H}\right)\right)}^{\beta }\right)}^{1/\beta },$$

with *β* = 1 − *α* = −2.583 for *α* = 3.583, a threshold min[*Int*(*aa*_*H*_)] = 4,000 nT⋅hr, and an assumed upper cutoff at max[*Int*(*aa*_*H*_)] = 21,000 nT⋅hr in rough agreement with GPD estimates (the negative *ξ* = −0.213 indicating the likely presence of an upper limit).

Figure 4b shows that the GPD and power‐law with upper cutoff distribution fits in Equations 4 and 5 yield very similar return levels, increasing with the number *n* of years until they reach a similar upper limit max[*Int*(*aa*_*H*_)] ≃ 20,000 nT⋅hr. The corresponding estimated return levels are close to the observed return levels over 1,868–2017. All the observed return levels are found within the 95% confidence intervals of the GPD and power‐law fits, except for one or two events (among max(*n*) = 150 in total) located slightly outside them. The highest return level (for a 95% confidence interval) of the GPD fit remains smaller than ∼22,500 nT⋅hr over 150 years. The upper limit estimate max[*Int*(*aa*_*H*_)] ≃ (min[*Int*(*aa*_*H*_)] − *σ*/*ξ*) ≈ 28,000 nT⋅hr provided by Extreme Value Theory is ∼40% higher than the 4 largest events observed in 1868–2017, which reached *Int*(*aa*_*H*_) = 18,400, 18,800, 19,300, 20,200 nT⋅hr in April 1994, October 2003, May 1921 (Love et al., 2019), and November 1882 (Love, 2018), respectively. These four events can be considered as typical 1 in 70 ± 40 years to 1 in 200 ± 130 years events. They agree well with the power‐law distribution fit with upper‐cutoff in Figure 4b. All these results suggest that *Int*(*aa*_*H*_) events larger than 21,000–22,500 nT⋅hr are unlikely to be observed in the next 50–150 years without an important change in the solar wind behavior. Let us caution, however, that the above estimates of return levels are based on the *Int*(*aa*_*H*_) distribution recorded in 1868–2017. Any forecast on this basis must further assume that this 150‐year distribution from the past will remain representative of future 10‐year to 150‐year distributions.

Unfortunately, no reliable estimate is available for the Carrington superstorm of September 1859, which has been estimated to have reached a slightly higher peak disturbance level in terms of *Dst* than the May 1921 superstorm (Cliver & Dietrich, 2013). Rough estimates (due to important data gaps) of the *aa* index during the Carrington storm available from the Helsinki magnetic observatory suggest a maximum daily‐averaged value only ∼15% higher than during four more recent storms, such as the November 1960 and March 1989 events (Nevanlinna, 2006). The estimated time‐integrated *Dst* was also probably weaker during this event than during more recent events, due to a fast recovery (Mourenas et al., 2018). Accordingly, the Carrington 1859 superstorm might not have exceeded the maximum *Int*(*aa*_*H*_) levels reached in 1868–2017.

### 3.3 Return Levels of High Time‐Integrated 2‐MeV Electron Flux Periods

**Figure 5:**
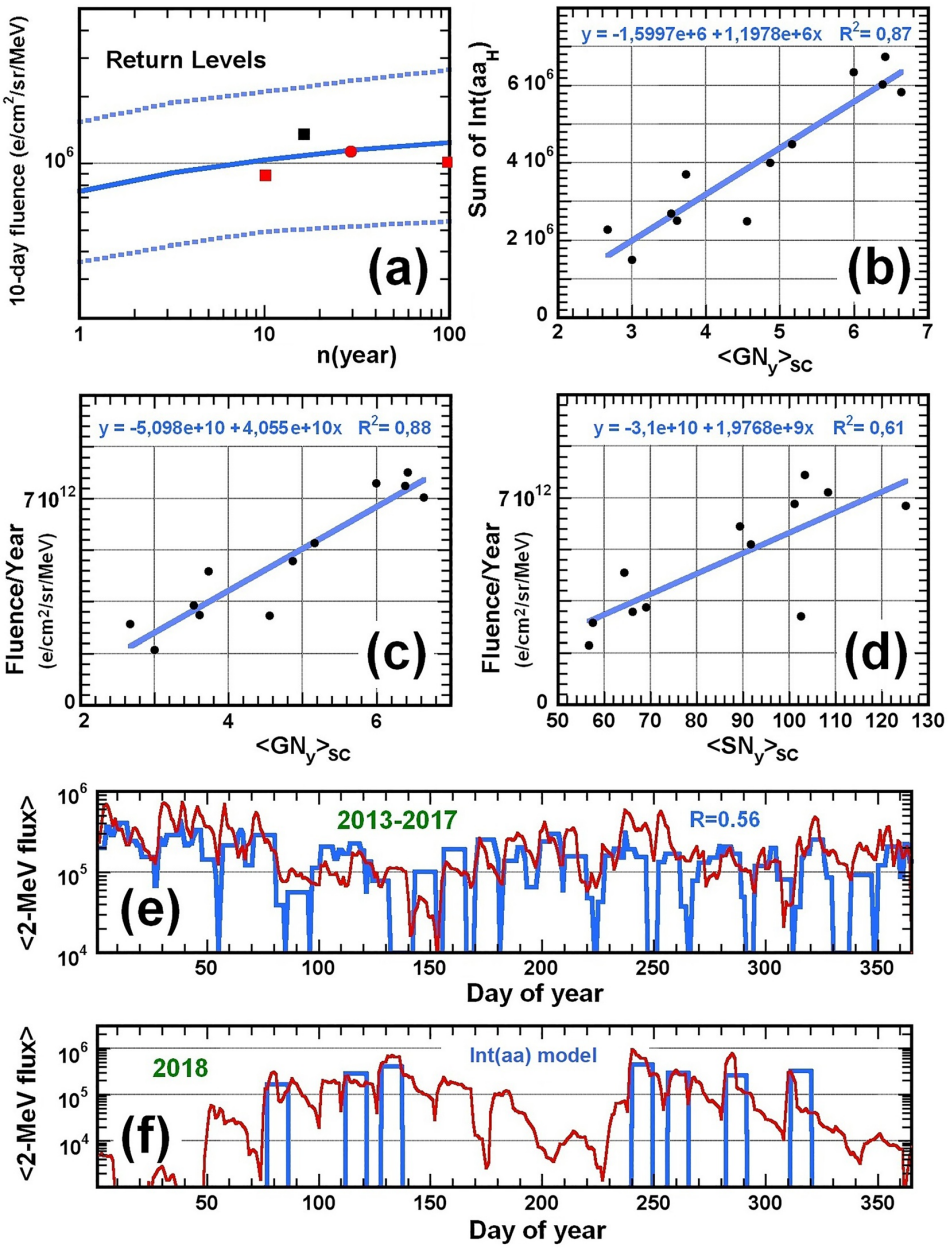
(a) Return levels of 10‐day‐integrated 2‐MeV electron flux (here in units of 10^6^ e/cm^2^/sr/MeV) at L* ∼ 4.5 (solid blue) based on ML power‐law fit in Equation 5, after a period of *n* years. Upper and lower bounds to these return levels are shown (dashed lines), as well as estimates based on a 14‐year statistics from INTEGRAL IREM (Meredith et al., 2017) at L* = 4.25 − 4.75 (red squares), the result (red circle) of a 30‐year Fokker‐Planck simulation at L* = 4.6 (Glauert et al., 2018), and the maximum level in 2001–2017 from Van Allen Probes and GPS satellite data (black square). (b) Variation of the sum of *Int*(*aa*_*H*_) > 1,400 nT⋅hr events as a function of the average yearly active‐day sunspot Group Number ${\langle GN_{y}\rangle }_{SC}$ during solar cycles in 1868–1995, with best fit. (c) Yearly time‐integrated 2‐MeV electron flux (i.e., fluence) over 10‐day periods following *Int*(*aa*_*H*_) > 1,400 nT⋅hr events at L* ∼ 4.5 based on the model, as a function of ${\langle GN_{y}\rangle }_{SC}$, with best fit. (d) Same as (c) but as a function of solar cycle‐averaged yearly International Sunspot Number ${\langle SN_{y}\rangle }_{SC}$ in 1868–2008. (e) Average daily 2‐MeV electron flux at L* ∼ 4.5 from the *Int*(*aa*_*H*_) model (blue) versus daily 2‐MeV electron flux measured by the Van Allen Probes in 2013–2017 (red), as a function of day of year. (f) Same as (e) for 2018, with model flux calculated from Equation 2 using *Int*(*aa*) instead of *Int*(*aa*_*H*_).

Figure 4b shows that the ML power‐law fit from Equation 5 gives $Int{\left(aa_{H}\right)}_{n}$ return levels very close to (albeit slightly higher than) GPD fit values. In addition, the 10‐day time‐integrated 2‐MeV electron flux increases only weakly (logarithmically) with *Int*(*aa*_*H*_) above 5,000 nT⋅hr in Figure 1b. Therefore, we can safely use the ML power‐law fit to estimate the return levels of time‐integrated 2‐MeV electron flux near L* ∼ 4.5. Upper and lower bounds corresponding to a ∼70% confidence interval are obtained by combining the maximum 95% confidence intervals of GPD and ML power‐law fits with the 70% confidence interval of the best fit in Figure 1b. Such estimated return levels of 10‐day time‐integrated 2‐MeV electron flux at L* ∼ 4.5 are displayed in Figure 5a. They are only weakly increasing with the number *n* of considered years (by a factor ∼1.7 from 1 to 100 years). 100‐year return levels of 10‐day‐integrated 2‐MeV electron flux reach 1.25 ⋅ 10^12^ e/cm^2^/sr/MeV at L* ∼ 4.5, with an upper bound estimated as ∼2.6 ⋅ 10^12^ e/cm^2^/sr/MeV.

Based on a 2002–2016 statistics from INTEGRAL IREM calibrated to MagEIS fluxes from the Van Allen Probes, Meredith et al. (2017) have also provided the return levels of daily 2‐MeV electron fluxes at L* ∼ 4.25 − 4.75, making use of Extreme Value Theory to extrapolate over a much longer 100‐year interval. Since the 2.1 MeV electron flux from REPT is on average ∼1.8 times larger than the corresponding MagEIS flux (Morley et al., 2016), and since such fluxes generally remain near their peak level for ∼7–10 days at L* = 4.5, we simply multiply by a factor 1.8 the daily flux return levels inferred from INTEGRAL IREM data and assume that they last 10 days to compare them with our results over 10‐day periods. Figure 5a shows that such return levels inferred directly from INTEGRAL IREM statistics are in good agreement with our estimated return levels at L* ∼ 4.5. A 30‐year Fokker‐Planck simulation utilizing measured electron fluxes from GOES as boundary condition (Glauert et al., 2018) also gives a similar maximum 10‐day‐integrated 2‐MeV electron flux at L* = 4.6 over 30 years.

It is well known that space weather approximately follows the solar cycle, with more frequent geomagnetic storms at solar maximum than solar minimum (Hathaway, 2015), higher 2–6 MeV electron fluxes in the outer radiation belt during the declining phase than near solar minimum (Baker et al., 2004). The most extreme daily‐averaged *aa*_*H*_ events are also more frequent during large solar cycles than small cycles (Owens et al., 2021). The average geomagnetic activity *aa* index is indeed correlated with sunspot number, albeit with significant scatter (Feynman, 1982). In Figure 5b, we find a high correlation (*R* ^2^ = 0.87) between the sum of *Int*(*aa*_*H*_) > 1,400 nT⋅hr values during a solar cycle and the corresponding average yearly active‐day sunspot Group Number *GN*_*y*_ (Usoskin et al., 2016). This high correlation can be used to estimate relativistic electron fluence during past or future solar cycles, based on past (Clette et al., 2014; Usoskin et al., 2016) or predicted (Petrovay, 2010) sunspot numbers. Figure 5c shows that the yearly time‐integrated 2‐MeV electron flux at L* ∼ 4.5 estimated using the *Int*(*aa*_*H*_) model in Equation 1 varies almost linearly with the average *GN*_*y*_ from solar cycle to solar cycle, with a similarly high correlation coefficient (*R* ^2^ = 0.88). Figure 5d shows a similar, albeit smaller, correlation (*R* ^2^ = 0.61) with the average yearly International Sunspot Number *SN*_*y*_ (Clette et al., 2014). Such results suggest a potential new way of estimating 2‐MeV electron fluence over the long term. However, comparisons with measurements would need to be performed over at least two different full solar cycles to validate this method. Moreover, a background of quiet‐day relativistic electron fluence should be added to the predicted active‐day fluence, although this quiet‐day fluence represents less than 25%–35% of the total fluence during years of moderate to high geomagnetic activity (Mourenas, Artemyev, & Zhang, 2019).

In the same vein, we show in Figure 5e the average daily 2‐MeV electron flux at L* ∼ 4.5 predicted by the *Int*(*aa*_*H*_) model and the daily 2‐MeV electron flux measured by the Van Allen Probes in 2013–2017, as a function of day of year. There is a significant correlation (*R* = 0.56) between modeled and measured average daily fluxes, demonstrating the model's capacity for providing good estimates of statistical seasonal and weekly variations of flux and time‐integrated electron flux.

Since the *aa*_*H*_ index is not (yet) available after 31 December 2017, it is important to check whether useful forecasts can still be provided after that date based on the proposed model, by simply using the non‐homogenized *aa* index (as readily available as *Kp*) instead of *aa*_*H*_ in Equation 2. Such a substitution is a priori justified, because differences between individual *aa* and *aa*_*H*_ values are small (<5–15%) and vary in sign during an event (Lockwood, Chambodut, Barnard, Owens, & Clarke, 2018; Lockwood, Chambodut, Barnard, Owens, Clarke, & Mendel, 2018), leading to even smaller differences between the time‐integrated *aa*_*H*_ and *aa* levels analyzed in the present study during strong events of continuously high *aa*_*H*_ ≥ 18 nT (or *aa* ≥ 18 nT). Figure 5f shows the daily 2‐MeV electron flux at L* ∼ 4.5 measured by the Van Allen Probes in 2018 (an out‐of‐sample year), compared to the modeled flux during the 10‐day periods that follow *Int*(*aa*) > 1,400 nT⋅hr events. The agreement between the *Int*(*aa*) model and measurements in 2018 remains similarly good as the agreement between the *Int*(*aa*_*H*_) model and measurements in 2017 (an in‐sample year) in Figure 2, with all the highest flux peaks exceeding 3 ⋅ 10^5^ e/cm^2^/sr/MeV/s well reproduced by the model. This confirms that both the *aa*_*H*_ index and the *aa* index can be used to provide useful predictions of time‐integrated 2‐MeV electron flux.

## 4 Conclusions

We developed a predictive model of long‐duration periods of high time‐integrated 2‐MeV electron flux near L* ∼ 4.5 deep inside the outer radiation belt, based on the significant correlation obtained between time‐integrated electron flux measured by the Van Allen Probes and GPS satellites in 2001–2017 and a peak‐over‐threshold measure (denoted *Int*(*aa*_*H*_)) of the preceding time‐integrated homogenized *aa*_*H*_ geomagnetic index. An analysis of four different events shows that this correlation is likely due to a stronger cumulative chorus wave‐driven acceleration of relativistic electrons and a stronger cumulative inward radial diffusion of such electrons by ULF waves during periods of higher time‐integrated geomagnetic activity. The predictive ability of the model has been assessed during both individual days and continuous 10‐day periods, using various skill scores and accuracy metrics, attesting its good efficiency in 2001–2017.

A key point of the present model is that the days of high 2‐MeV electron flux (and time‐integrated flux) are predicted, on average, 5 days in advance (between 0 and 10 days ahead). This suggests that this simple but efficient model could be used as a complement to other, more sophisticated forecast models (e.g., Glauert et al., 2021; Pires de Lima et al., 2020) for providing a far‐ahead warning of dangerous long‐duration periods of particularly elevated time‐integrated relativistic electron flux that should be examined in more detail. In addition, the present model only relies on the past history of a unique *aa*_*H*_ ground‐based geomagnetic index. We showed that the *aa* index can be used instead of *aa*_*H*_ in the model, thanks to small differences between time‐integrated *Int*(*aa*_*H*_) and *Int*(*aa*) parameters. Although this model has been developed for 2‐MeV electron flux because it represents an important risk for spacecraft electronics, electron flux energy spectra are often coherent over ∼1.5–3 MeV during prolonged periods of high flux, suggesting that the model could be applied over this whole energy range, using typical energy spectral shapes observed during such periods of high flux. At lower and higher energy, however, the presence of electron injections and the different time scales of electron acceleration would require to develop other specific models.

Return levels of 2‐MeV electron flux have been provided based on Extreme Value analysis of time‐integrated geomagnetic activity over 1868–2017. Let us caution, however, that such forecasts need to assume that the analyzed 150‐year distribution of *aa*_*H*_ is representative of future distributions and that the same correlations between peaks of *Int*(*aa*_*H*_) and peaks of time‐integrated 2‐MeV electron flux will remain valid in the future. Notwithstanding these limitations, the provided maximum return levels of 10‐day time‐integrated 2‐MeV electron flux roughly agree with previous independent studies based on statistical analyses of <20‐year data sets of measured electron fluxes. The maximum 10‐day‐integrated flux of 2‐MeV electrons at L* ∼ 4.5 is estimated as ≃ 1.25 ⋅ 10^12^ e/cm^2^/sr/MeV for a 1 in 100 years event. Extreme Value theory suggests that *Int*(*aa*_*H*_) events probably have an upper limit, which could be related to various physical mechanisms of adaptation of the magnetosphere to strong and prolonged solar wind impacts. Steep dropouts of electron flux due to magnetopause shadowing should also limit time‐integrated 2‐MeV electron flux between *L* ∼ 4.2 and geostationary orbit, with much more frequent dropouts occurring at higher *L* (Boynton et al., 2017; Boynton, Mourenas, & Balikhin, 2016).

Finally, we found a high correlation between the *Int*(*aa*_*H*_) measure of time‐integrated geomagnetic activity averaged over each solar cycle and the similarly averaged International Sunspot Number and active‐day sunspot Group Number. This suggests that forecasts of time‐integrated relativistic electron flux during future solar cycles might be obtained based on predictions of future solar activity.

## Figures and Tables

**Figure A1:**
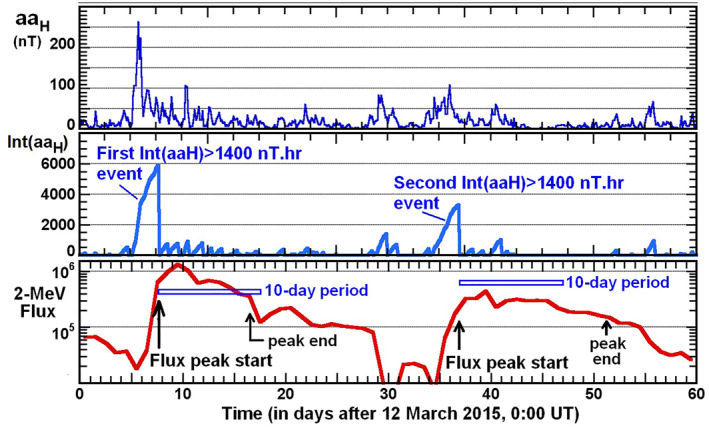
Two *Int*(*aa*_*H*_) > 1,400 nT⋅hr events in March–May 2015. (top) *aa*_*H*_ (in nT) index as a function of time. (Middle) *Int*(*aa*_*H*_) (in nT⋅hr), calculated by summing hourly *aa*_*H*_ values as long as *aa*_*H*_ remains continuously ≥18 nT. (Bottom) daily 2‐MeV electron flux (in e/cm^2^/s/sr/MeV) measured by the Van Allen Probes at L* ∼ 4.5. The start and end of flux peaks are indicated by black arrows. The considered 10‐day periods of high time‐integrated flux starting immediately after each *Int*(*aa*_*H*_) > 1,400 nT⋅hr event are marked by blue rectangles.

**Figure B1:**
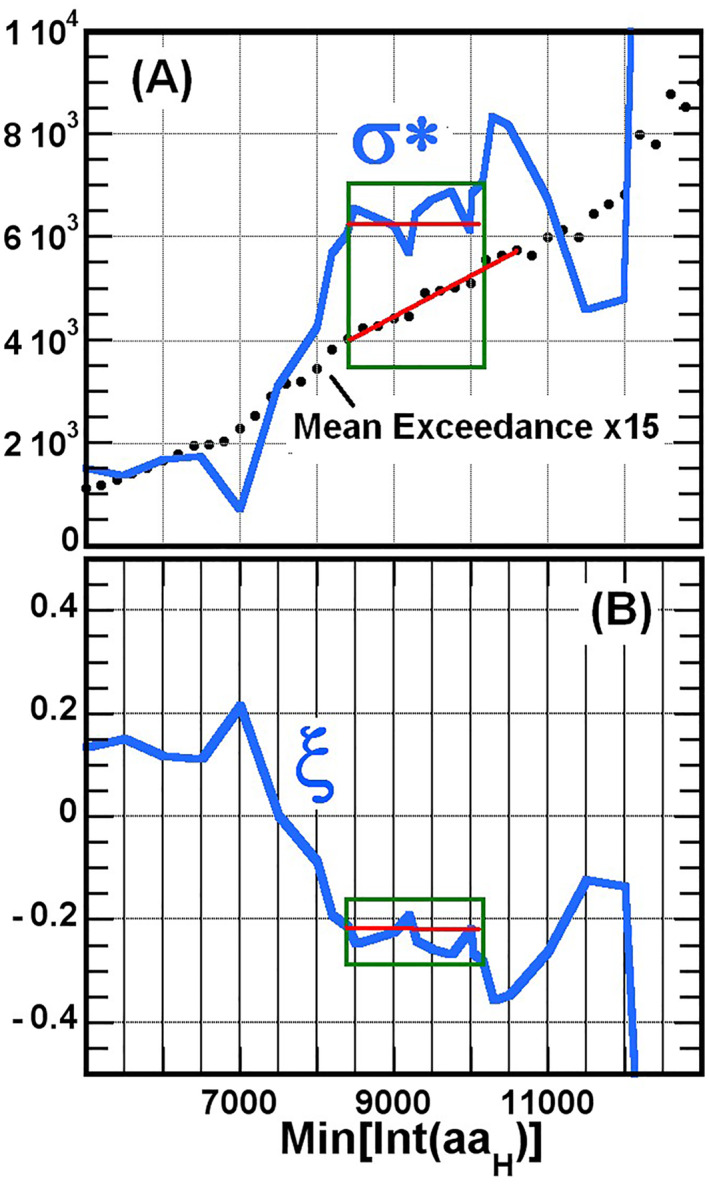
(a) Mean exceedance 〈*Int*(*aa_H_*) − min[*Int*(*aa_H_*)]〉 (black points) and σ* (blue) as a function of the threshold min[*Int*(*aa_H_*)] in 1868–2017. (b) *ξ* GPD parameter (blue) as a function of the threshold min[*Int*(*aa_H_*)] in 1868–2017. Green boxes show the domain of simultaneously nearly constant σ* and *ξ* and linearly increasing mean exceedance.

## Data Availability

Van Allen Probes REPT electron flux data (REL03 L2) is available from NASA at https://cdaweb.gsfc.nasa.gov/cgi-bin/eval1.cgi, and LANL CXD data of GPS electron flux (2017 release) is available from NOAA at https://www.ngdc.noaa.gov/stp/space-weather/satellite-data/satellite-systems/gps/. The *aa*_*H*_ index can be retrieved at https://www.swsc-journal.org/articles/swsc/olm/2018/01/swsc180022/swsc180022-2-olm.txt. The *aa* index is calculated by ISGI collaborating institutes from data collected at magnetic observatories and available at http://isgi.unistra.fr. The OMNI data of *AE*, *Kp*, and *Dst* are available from the GSFC/SPDF OMNIWeb interface at https://omniweb.gsfc.nasa.gov. The active‐day Sunspot Group Number and International Sunspot Number V2.0 are available from SILSO (Royal Observatory of Belgium, Brussels) at http://sidc.be/silso/.

## Appendix A Examples of *Int*(*aa*_*H*_) Events

Two typical examples of *Int*(*aa*_*H*_) > 1,400 nT⋅hr events are displayed in Figure A1 between 12 March and 10 May 2015. The top panel shows the variation of the *aa*_*H*_ index with time, while the middle panel shows the level of *Int*(*aa*_*H*_), integrated over time as long as *aa*_*H*_ remains continuously higher than 18 nT. The bottom panel shows the corresponding daily 2‐MeV electron flux measured by the Van Allen Probes at *L** = 4.5. Blue rectangles denote the 10‐day periods of high flux considered in this study, which start at the end of each *Int*(*aa*_*H*_) > 1,400 nT⋅hr event. Black arrows indicate the start and end of actual flux peaks, determined at 1/2 and 1/3 of the maximum flux.

## Appendix B Determination of GPD Fit Parameters

Here, we provide a brief description of the derivation of appropriate scale and shape parameters of the GPD distribution fit shown in Figure 4. The appropriate threshold min[*Int*(*aa*_*H*_)] for a reliable GPD fit must correspond to (a) a sufficiently high min[*Int*(*aa*_*H*_)] in the distribution tail (usually within the 60 upper points), (b) a linear relationship between mean exceedance 〈*Int*(*aa*_*H*_) − min[*Int*(*aa*_*H*_)]〉 and min[*Int*(*aa*_*H*_)] over some range of min[*Int*(*aa*_*H*_)], and (c) nearly stable (constant) estimates of GPD parameters *ξ* and *σ** = *σ* − *ξ* ⋅ min[*Int*(*aa*_*H*_)] obtained via the Maximum Likelihood (ML) method over the same min[*Int*(*aa*_*H*_)] range (Coles, 2001; Tsubouchi & Omura, 2007). In Figures B1a and B1b, the mean exceedance (black points) shows some evidence of linearity over the same domain min[*Int*(*aa*_*H*_)] ≃ 8,400–10,150 nT⋅hr (corresponding to the 44 upper data points) where *ξ* and *σ** are simultaneously nearly constant in Figures B1a and B1b.

The optimal (*ξ*, *σ*) parameters generally correspond to the lowest appropriate threshold min[*Int*(*aa_H_*)], because more events are then taken into account, which decreases uncertainties (Coles, 2001). Here, it gives min[*Int*(*aa_H_*)] = 8,400 nT⋅hr, corresponding to a shape parameter *ξ* ≃ −0.213 ± 0.33 and a scale parameter *σ* ≃ 4,260 ± 1,880 (a higher min[*Int*(*aa_H_*)] = 9,300 nT⋅hr gives similar *ξ* = −0.24 ± 0.398 and *σ* ≃ 4,230 ± 2160 values but with larger uncertainties), where minimum and maximum parameter values correspond to 95% confidence intervals calculated via the delta method (Coles, 2001). This GPD fit is shown in Figure 4a.
